# The Human RNA Helicase DDX21 Presents a Dimerization Interface Necessary for Helicase Activity

**DOI:** 10.1016/j.isci.2020.101811

**Published:** 2020-11-18

**Authors:** Maria J. Marcaida, Annamaria Kauzlaric, Alice Duperrex, Jenny Sülzle, Martin C. Moncrieffe, Damilola Adebajo, Suliana Manley, Didier Trono, Matteo Dal Peraro

**Affiliations:** 1 Institute of Bioengineering, School of Life Sciences, Ecole Polytechnique Fédérale de Lausanne (EPFL), Lausanne, 1015 Switzerland; 2 Global Health Institute, School of Life Sciences, Ecole Polytechnique Fédérale de Lausanne (EPFL), Lausanne, 1015 Switzerland; 3 Laboratory for Experimental Biophysics, Institute of Physics, Ecole Polytechnique Fédérale de Lausanne (EPFL), Lausanne, 1015 Switzerland; 4 Department of Biochemistry, University of Cambridge, Tennis Court Road, Cambridge CB2 1GA, UK

**Keywords:** Molecular Modelling, Biological Sciences, Molecular Biology, Structural Biology

## Abstract

Members of the DEAD-box helicase family are involved in all fundamental processes of RNA metabolism, and as such, their malfunction is associated with various diseases. Currently, whether and how oligomerization impacts their biochemical and biological functions is not well understood. In this work, we show that DDX21, a human DEAD-box helicase with RNA G-quadruplex resolving activity, is dimeric and that its oligomerization state influences its helicase activity. Solution small-angle X-ray scattering (SAXS) analysis uncovers a flexible multi-domain protein with a central dimerization domain. While the Arg/Gly rich C termini, rather than dimerization, are key to maintaining high affinity for RNA substrates, *in vitro* helicase assays indicate that an intact dimer is essential for both DDX21 ATP-dependent double-stranded RNA unwinding and ATP-independent G-quadruplex remodeling activities. Our results suggest that oligomerization plays a key role in regulating RNA DEAD-box helicase activity.

## Highlights

• The human RNA DEAD-box helicase DDX21 is dimeric
• DDX21 dimerization is mediated by a hydrophobic central core domain
• SAXS-based modeling reveals that DDX21 is intrinsically flexible
• Dimerization and C-terminal domains mediate G-quadruplex and dsRNA unwinding

## Graphical Abstract

**Figure:**
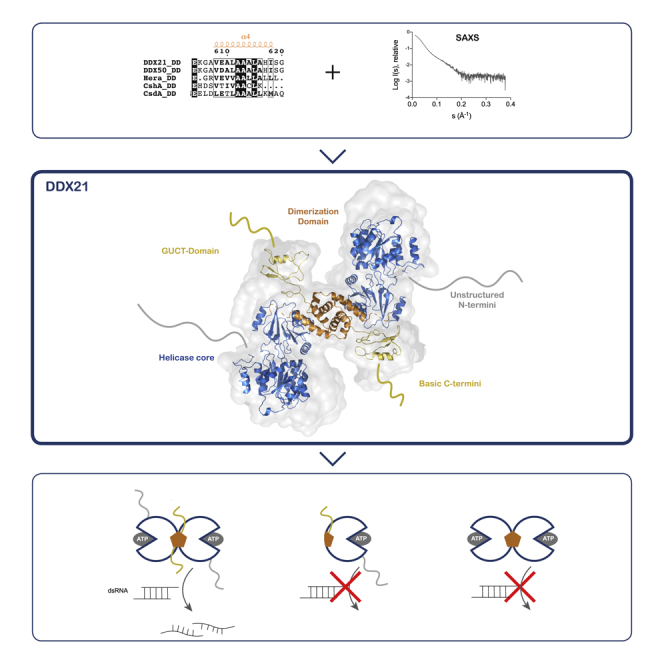

## Introduction

RNA DEAD-box helicases perform structural rearrangements of RNA and RNA/protein complexes in many cellular processes including ribosome biogenesis, transcription, translation, and RNA editing (Linder and Jankowsky, 2011). Their names are derived from a characteristic sequence of amino acids (D-E-A-D) that is involved in ATP binding. Members of the DEAD-box family are present in all domains of life and share a common helicase core (HC) composed of two domains homologous to the bacterial single-stranded DNA-binding protein, RecA (Story et al., 2001) (Figure S1). A flexible linker connects the two RecA-like domains (RecA-N and RecA-C). The HC is able to couple the chemical energy from ATP binding and hydrolysis to unwind double-stranded RNA (dsRNA) in a non-processive manner, thanks to several conserved short motifs that mediate either ATP or RNA binding, or intramolecular interactions. The flexible linker allows for large variations in the orientation and distance of the RecA-like domains with respect to each other during the catalytic cycle. Some of these conformations have been observed in crystal structures, depicting the unwinding reaction: the apo, pre-unwound, and post-unwound states (Chen et al., 2020; Henn et al., 2012; Ozgur et al., 2015). In the apo state, the two RecA-like domains are in the open conformation without a defined relative orientation. In the pre-unwound state, dsRNA binding causes structural rearrangements and domain closure (Mallam et al., 2012; Song and Ji, 2019). Further dramatic changes take place to unwind the duplex, resulting in the post-unwound state where one HC is bound to ATP and single-stranded RNA (ssRNA), as seen, for example, for the enzyme Vasa and for DDX21 (Chen et al., 2020; Sengoku et al., 2006) (Figure S1). For some enzymes, strand separation requires only ATP binding, while ATP hydrolysis causes the release of RNA and the recycling of the enzyme (Liu et al., 2008). Despite the high level of structural and sequence conservation, the nucleotide-dependent RNA-binding mode and the modular function of the RecA-like domains are not universal and can vary among DEAD-box proteins (Samatanga and Klostermeier, 2014). Similarly, the oligomerization state of the enzyme on its own or bound to RNA is a factor that varies among family members and could even change during the catalytic cycle. Therefore oligomerization needs to be considered when studying the mechanism of action of DEAD-box helicases, but it has thus far been investigated only for a few members of the family (Huen et al., 2017; Klostermeier, 2013; Minshall and Standart, 2004; Ogilvie et al., 2003; Putnam et al., 2015; Song and Ji, 2019; Xu et al., 2017).

In many cases, DEAD-box helicases also contain additional N- or C- terminal domains that contribute to higher enzymatic efficiency or mediate specific protein/RNA interactions (Del Campo et al., 2009; Grohman et al., 2007; Jarmoskaite and Russell, 2014; Linden et al., 2008; Mallam et al., 2011; Mohr et al., 2008). DEAD box proteins in general display little sequence specificity for RNA, although in some examples, the accessory domains recognize specific RNA structural elements (Kossen et al., 2002; Steimer et al., 2013). The human DEAD-box RNA helicase DDX21, also known as nucleolar RNA helicase II/Guα, falls into this latter class. DDX21 can bind and remodel RNA G-quadruplexes using amino acids at the C terminus of the protein, outside the HC (McRae et al., 2017, 2018). DDX21 is involved in multiple steps of ribosome biogenesis like rRNA processing (Henning et al., 2003) and the recruitment of small nucleolar RNAs into pre-ribosome complexes (Sloan et al., 2015). Furthermore, it has been implicated in transcription by RNA Polymerase I and II, through direct binding of transcription factors like c-Jun (Holmström et al., 2008; Westermarck et al., 2002; Zhang et al., 2014) or through formation of large RNA/protein complexes with long non-coding RNAs such as 7SK or SLERT (Calo et al., 2015; Xing et al., 2017). It is also one of the human DEAD-box helicases implicated in viral infection (Dong et al., 2016; Hammond et al., 2018; Naji et al., 2012; Zhang et al., 2011). For example, DDX21 inhibits influenza A viral replication by inhibiting the viral RNA polymerase (Chen et al., 2014), but it is required for efficient HIV viral production via Rev-related mechanisms (Hammond et al., 2018; Naji et al., 2012). This broad spectrum of functions might reflect the fact that DDX21, in addition to being a *bona fide* helicase able to unwind dsRNA, can also resolve other nucleic acid structures like R-loops or RNA G-quadruplexes (Argaud et al., 2019; Hao et al., 2019; McRae et al., 2017, 2018, 2020; Song et al., 2017). R-loops are cellular structures composed of a ssRNA:dsDNA hybrid that are dynamically formed and resolved at specific loci in association with different transcription phases (Santos-Pereira and Aguilera, 2015). When they form, the exposed ssDNA can be susceptible to DNA damage, and therefore mechanisms that resolve R-loops are important to maintain genomic integrity. Similarly, G-quadruplexes are also non-canonical nucleic acid secondary structures found in sequences rich in guanine residues. Resolving such structures in a timely and regulated manner is key to maintaining genome stability and thus avoiding certain diseases (Agarwala et al., 2015; Santos-Pereira and Aguilera, 2015). Indeed, DDX21 dysregulation has been observed in multiple human cancers (Kim et al., 2019; Zhang et al, 2014, 2018) and was linked to developmental disorders (Calo et al., 2018). Despite growing evidence of its prominent roles, there is still very little known about how the helicase uses its various auxiliary domains to modulate the unwinding and remodeling of the different RNA substrates, thus regulating its function.

This work provides the first structural and biochemical characterization of the accessory domains of DDX21, revealing that it is a homodimer and identifying its dimerization interface. Because of the inherent flexibility of the multi-domain organization of DDX21, we used an integrative approach (Degiacomi et al., 2013; Fonti et al., 2019; Tamò et al., 2015) combining biochemical and biophysical analyses as well as solution small-angle X-ray scattering (SAXS) data and flexible fitting. In light of our results, we propose that an intact dimerization interface is essential for maintaining two DDX21 activities: dsRNA unwinding and G-quadruplex remodeling. We further discuss the implications of oligomerization for the current understanding of the modular function of DEAD-box helicases.

## Results

### DDX21 Is a Multi-Domain Homodimer

**Figure 1:**
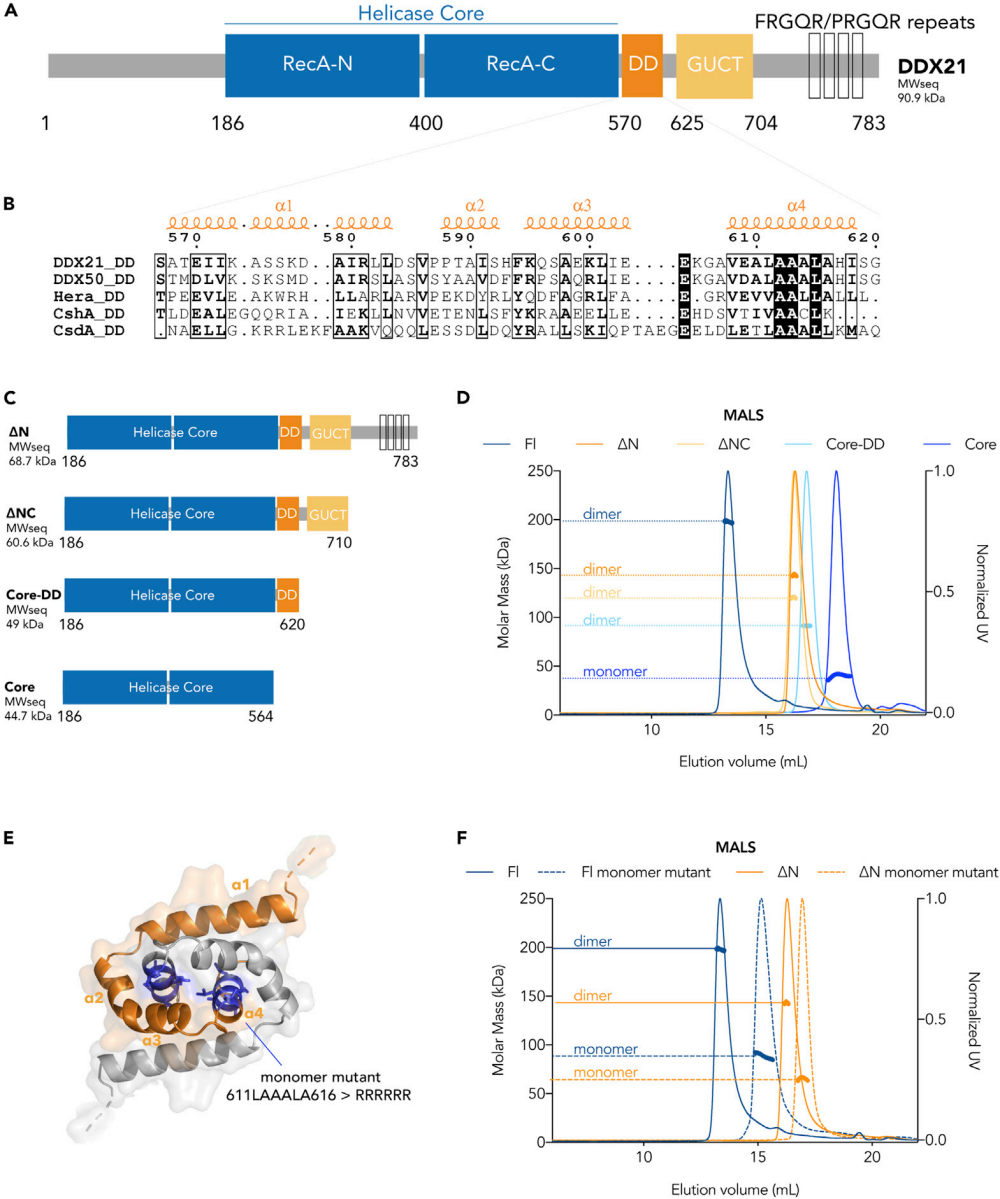
Identification of the Dimerization Domain of DDX21 (A) Numbered linear sequence with the different domains colored in blue (HC), orange (DD), and yellow (GUCT) and the C-terminal tail with the FRGQR/PRGQR repeats in black boxes. The molecular weight of this sequence is MW_seq_ = 90.9 kDa (cleaved affinity tag) (see also Figures S1, Figure 1, Figure 2, Figure 3, Figure 4, Figure 5and S7). (B) Sequence alignment of the DDs from the two human paralogs DDX21 and DDX50, as well as the bacterial dimeric DEAD-box helicases Hera, CshA, and CsdA (see also Figure S3). (C) Schematic representation of the truncation constructs used in this study with their corresponding monomeric MW_seq_ calculated from their sequences. (D) SEC-MALS data show that all the constructs except DDX21_Core_ are dimers (theoretical molar masses [MW_seq_] for Fl, ΔN, ΔNC, Core-DD, and Core are 90.9, 68.7, 60.6, 49, and 44.7 kDa, respectively). Experimental SEC-MALS molar masses are Fl, 198 kDa; ΔN, 143 kDa; ΔNC, 120 kDa; Core-DD, 91 kDa; and Core, 41 kDa) (see also Figures S4 and S5). (E) Homology model of the DD of DDX21 in cartoon representation, with helices α1 to α4 of each protomer colored in orange and gray, respectively. The residues in α4 mutated to create the monomeric mutants are shown in blue stick representation. (F) SEC-MALS of the Fl and ΔN monomeric mutants (in dashed lines) with molar masses of 88 and 66 kDa, compared with intact DDX21_Fl_ (blue) and DDX21_ΔN_ (orange) with molar masses of 198 and 143 kDa.

Previous work has shown that in addition to the DEAD-box HC, DDX21 contains a Gu C-terminal (GUCT) domain and a C-terminal basic tail (also described as an RGG/RG motif containing four repeats of the sequence FRGQR/PRGQR, Figure 1A) (Flores-Rozas and Hurwitz, 1993; Valdez, 2000). All domains are necessary for HeLa cell growth and cell cycle progression (Ou et al., 1999). The GUCT domain adopts the fold of an RNA recognition motif (RRM) (Daubner et al., 2013) with a central four-stranded β-sheet and two α-helices (Figure S2) (PDB code 2M3D, unpublished). Comparison of the electrostatic potential distribution on the surface of the GUCT/RRM domains from different enzymes might suggest they have different functions and will be discussed later in the context of RNA binding.

Structure-based sequence alignments and homology modeling template searches have allowed us to identify a putative dimerization domain (DD) (residues 568–620) (Figure 1B). The templates used are three dimeric bacterial proteins (Huen et al., 2017; Klostermeier, 2013; Xu et al., 2017) whose sequence alignment is shown in Figures 1B and S3. On the basis of the structural inferences derived from the three bacterial homodimeric helicases, we hypothesized that the DD in human DDX21 also drives self-association, as is the case for the bacterial enzymes. Indeed, when full-length DDX21 (DDX21_Fl_) and several N- and C-terminal truncation constructs were expressed and purified (Figure 1C), they behaved as dimers, as shown by multi-angle light scattering coupled to size exclusion chromatography (SEC-MALS) experiments (Figure 1D). On the contrary, as expected, the HC by itself is monomeric (Figure 1D). DDX21_Fl_ dimerization was further confirmed by cross-linking and analytical ultracentrifugation equilibrium sedimentation experiments (Figure S4).

In the bacterial structures, the DD consists of four α-helices that each pack compactly with the corresponding helices of the dimeric partner, forming the so-called “knot” that is stabilized by hydrophobic and hydrogen bonding interactions (Huen et al., 2017; Klostermeier and Rudolph, 2009; Xu et al., 2017). Knots are rare but have been reported for the DD of the three bacterial helicase crystal structures, and the pattern of amino acids that support the dimers of the bacterial proteins also occurs in the human DDX21 DD (Figure 1B). Based on these templates, a model for the DD of DDX21 was generated (Figure 1E). The homology model suggests that the hydrophobic core of the DD forms the main hydrophobic interface of the dimer. This is composed of residues of the two α4 helices, specifically residues 608 to 616. To test this hypothesis, DDX21_Fl_ and DDX21_ΔN_ mutants were generated in which the conserved hydrophobic residues in the α4 helices (residues 611–616 [LAAALA]) were mutated to charged arginines, to create repulsion upon dimerization (Figures 1B and 1E). The two mutants were expressed, purified, and well folded, as were all the other DDX21 constructs (confirmed by circular dichroism, Figure S5). Both DDX21 mutants behaved as monomers in SEC-MALS experiments (Figure 1F), confirming that DDX21 dimerization requires the hydrophobic interface provided by the α4 helices in the DD to be intact.

### DDX21 Binds R-Loops as a Dimer

**Figure 2:**
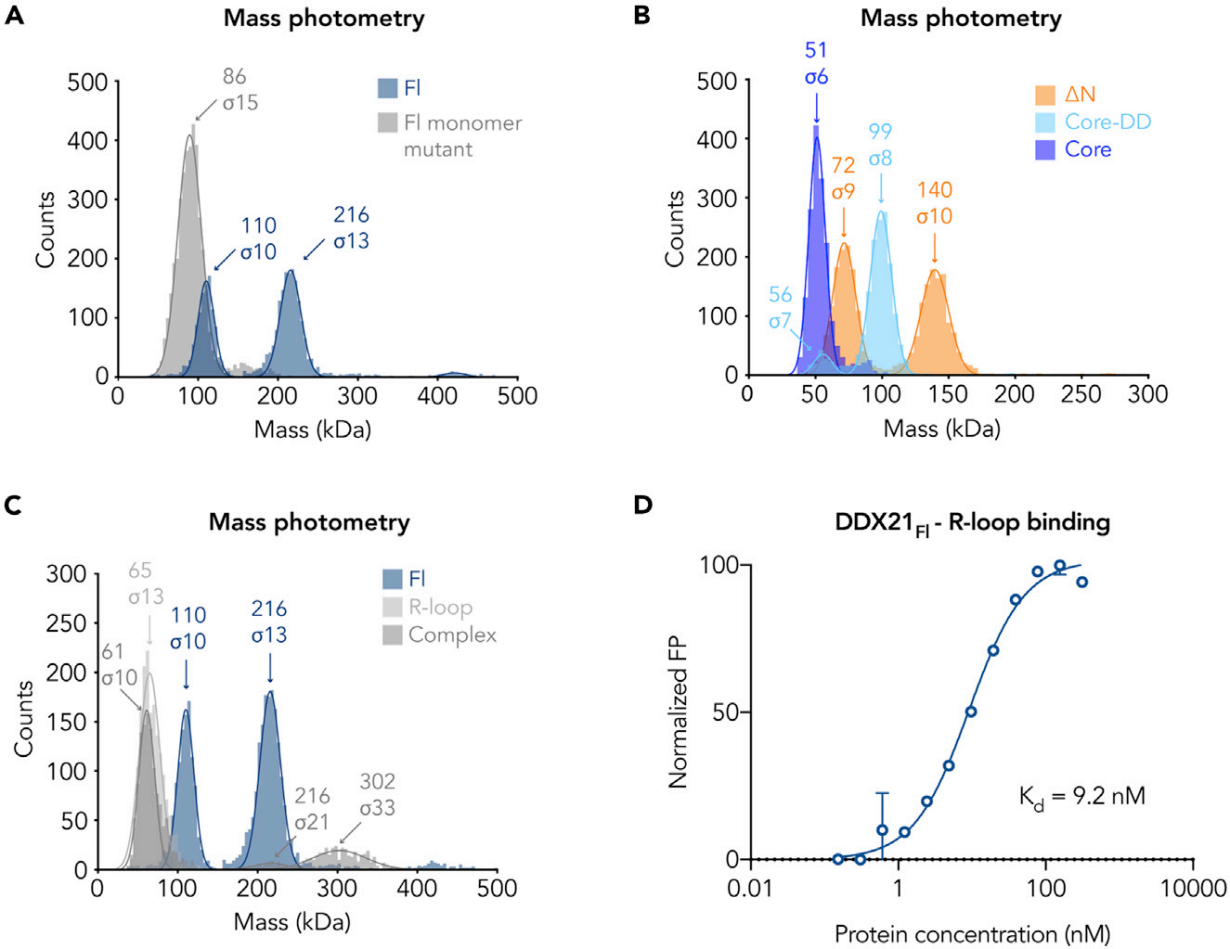
The DDX21 Dimer Binds Two R-loop Molecules (A) Mass photometric profiles obtained for DDX21_Fl_ (in blue) and DDX21_Fl_ monomer mutant (in gray) with the determined average molecular mass indicated above each peak. The theoretical molar mass for DDX21_Fl_ (with uncleaved affinity tag) is 104.9 kDa and for the DDX21_Fl_ monomer mutant is 90 kDa. The wild-type protein shows a monomer-dimer equilibrium, whereas the mutant is mainly monomeric. (B) Mass photometric profiles for DDX21_ΔN_, DDX21_Core-DD_, and DDX21_core_ indicate that DDX21_ΔN_ displays a monomer-dimer equilibrium as seen for DDX21_Fl_, whereas DDX21_Core-DD_ and DDX21_core_ are mainly dimeric and monomeric, respectively. (C) Mass photometric profile for the DDX21_Fl_-R-loop complex at 30 nM. (D) Fluorescence polarization binding assays measure a binding affinity of DDX21_Fl_ to the R-loop substrate of 9.2 ± 0.7 nM. Error bars represent the standard deviation of three independent measurements. The mass photometric experiments were performed in duplicates for the protein samples and in triplicates for the R-loop complex (see also Figure S6).

We next investigated the oligomeric state of DDX21 in the presence of RNA. The protein-RNA complexes were unstable under the conditions suitable for SEC-MALS analysis; therefore we used mass photometry to estimate the molecular mass of the macromolecular assemblies in solution (Sonn-Segev et al., 2020). The basis of mass photometry is the optical detection of the scattering signal generated by a single particle at the glass-water interface. This signal scales linearly with the mass of the particle. After calibrating the signal with a sample of known mass, mass photometry accurately provides mass distributions. The histograms in Figures 2A–2C show the measured distribution of molecular mass estimates for individual DDX21 macromolecular complexes and RNA molecules. Because mass photometry is limited to single events, the concentration of macromolecules is limited to the low nanomlolar range. Thus, we used 30 and 15 nM, respectively. DDX21_Fl_ was detected as two peaks of 110 ± 10 and 216 ± 13 kDa, whereas the monomer mutant showed only one peak at 86 ± 15 kDa (Figure 2A). This indicates that at this low concentration, DDX21_Fl_ displays a monomer-dimer equilibrium, whereas the mutant is mainly monomeric. Further mass photometric analysis of the truncation mutants corroborated the molecular masses measured by MALS: both DDX21_ΔN_ and DDX21_Core-DD_ display two peaks that are consistent with a monomer-dimer equilibrium, whereas the system without the DD (DDX21_Core_) is mainly monomeric (Figure 2B).

As a nucleic acid substrate we choose the R-loop described by Song and colleagues (Song et al., 2017), because its molecular weight (45 kDa) allows for the mass change upon complex formation to be detectable by this technique. As shown by fluorescence polarization (FP) binding experiments, purified DDX21_Fl_ binds the R-loop substrate with nM affinity (9.2 ± 0.7 nM, Figure 2D), which is within the affinity range suitable for mass photometric analysis. The R-loop alone was detected as a single peak at 65 ± 13 kDa. This difference is likely due to the different refractive indices and charges of proteins and nucleic acids and the fact that 45 kDa is at the low mass detection limit of the technique. The DDX21_Fl_-R-loop complex at 30 nM was detected as three peaks: the first peak corresponds to excess R-loop (61 ± 10 kDa), the second peak to the protein dimer (216 ± 21 kDa), and the third peak to a protein dimer bound to two R-loop molecules (302 ± 33 kDa) (Figures 2C and S6).

These experiments suggest that DDX21 is a dimer in the apo conformation as well as in complex with RNA substrates. DDX21 appears therefore structurally homologous to the bacterial enzymes (i.e., Hera, CshA, and CsdA) and dimerizes via a small hydrophobic helical domain. Inspection of its DD amino acid composition suggests that the DDX21 paralog DDX50 might also be a dimer (Figures 1B and S7). Altogether, we identified a previously unknown dimerization domain for DDX21 yet potentially important for helicase activity.

### DDX21 Dimerization Is Essential for dsRNA Unwinding

**Figure 3:**
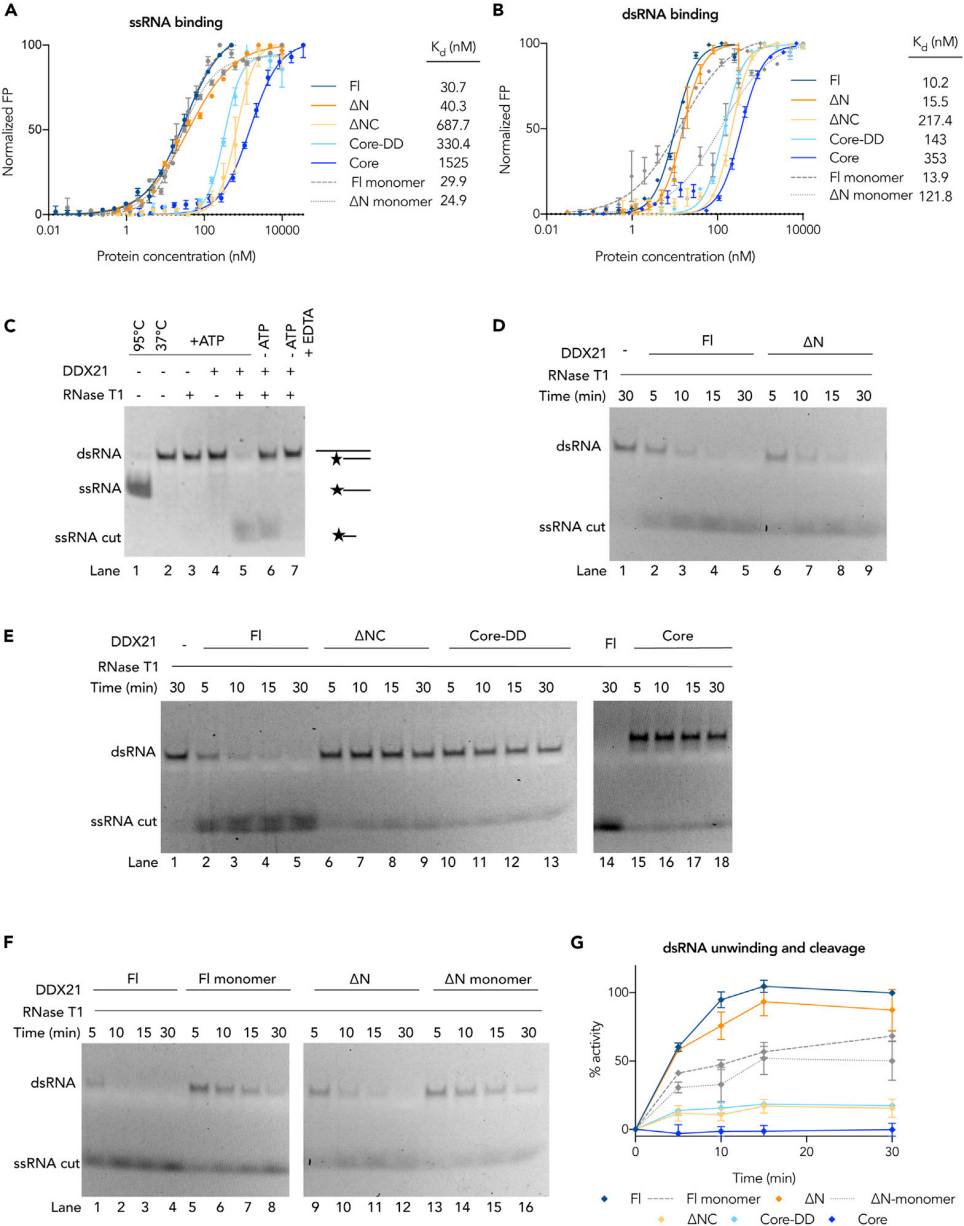
dsRNA Binding and Unwinding by DDX21 (A and B) FP experiments comparing the binding affinity of the DDX21 mutants to the (A) 15-mer ssRNA and the (B) dsRNA with a 3′ 15-base overhang, respectively. Error bars represent the standard deviation of three independent measurements (see also Table S1). (C) dsRNA helicase assay control experiment (time = 5 min) showing that upon addition of ATP, DDX21 can unwind the dsRNA, such that the exposed ssRNA can be cut by RNase T1 (lane 5). Without DDX21, RNase T1 cannot cut the substrate (lane 3). As described by Hammond et al. (2018) and Valdez et al. (1997) in the absence of ATP (lane 6), DDX21 has some residual helicase activity, which disappears upon addition of 5 mM EDTA (lane 7). The nature of the RNA bands in the gel is depicted on the right, where the star represents the 5′FAM modification. (D–F) dsRNA helicase assay comparing the activity of the different DDX21 mutants at different time points (5, 10, 15, and 30 min). In native condition polyacrylamide gels, the 5′-FAM-labeled RNA was visualized by measuring the fluorescent signal at 535 nm. (G) The helicase assays shown in (D–F) were repeated in triplicate (see also Figure S8); the activity was calculated by measuring the intensity of the product (cut) RNA bands, with respect to DDX21_Fl_ activity; and the quantification is shown in the graph, where the points represent the mean of three independent measurements and the error bars represent the standard error.

**Figure 4:**
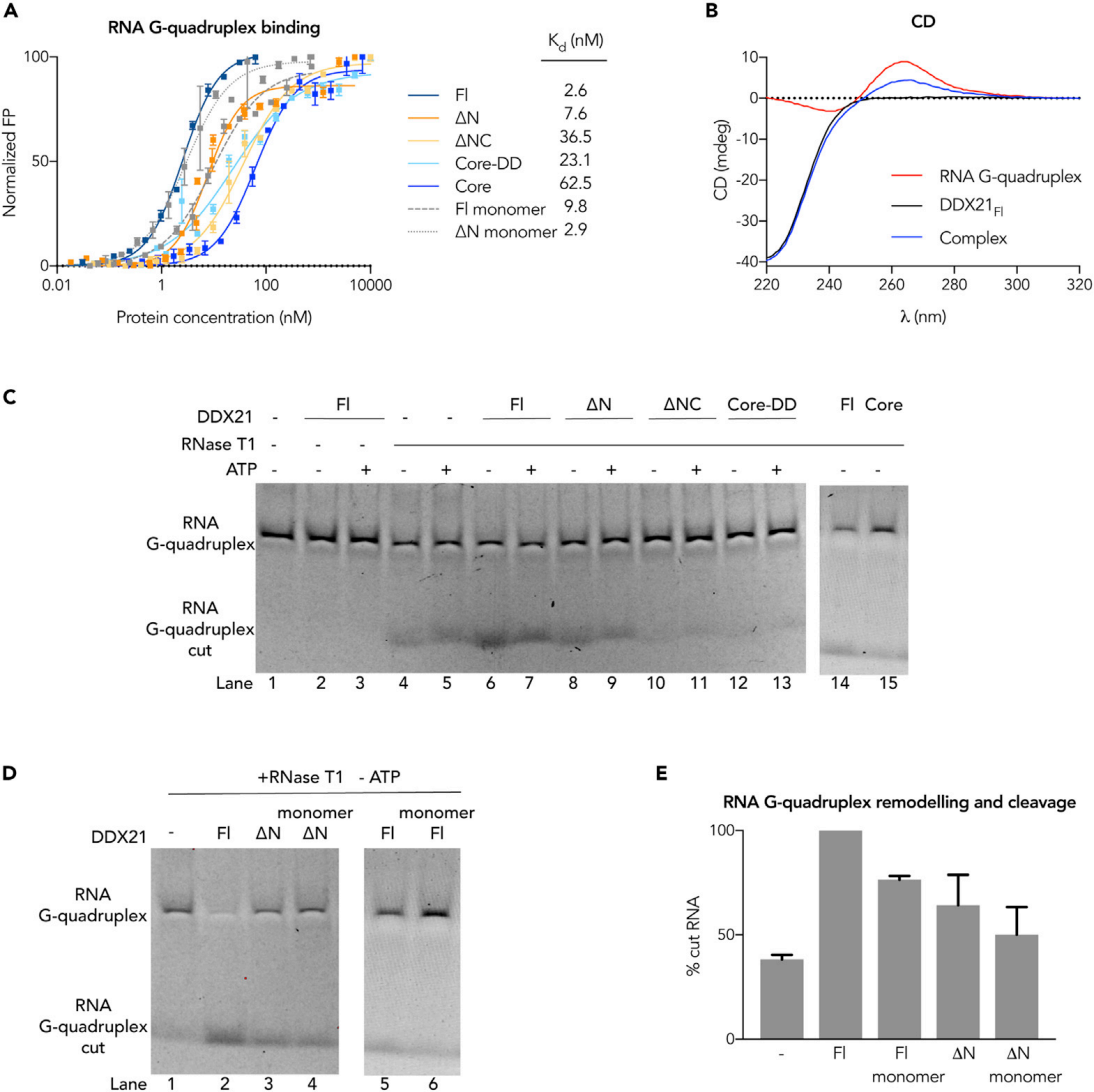
DDX21 Activity on RNA G-Quadruplexes (A) FP binding curves for the different DDX21 variants and the RNA G-quadruplex Q2. Error bars represent the standard deviation of three independent measurements (see also Figure S9 and Table S2). (B) The circular dichroism (CD) spectra of the RNA G-quadruplex (in red) with a parallel structure with its characteristic maximum at 260 nm and minimum at 240 nm. In the presence of DDX21_Fl_ (blue), this structure is maintained, as showed by the maximum at 260 nm that is not present in the spectrum of the protein alone (black) (see also Figure S10). (C and D) RNA G-quadruplex remodeling assay comparing the activity of the different DDX21 mutants. In native condition polyacrylamide gels, the 5′-FAM-labeled RNA was visualized by measuring the fluorescent signal at 535 nm. The activity was calculated by measuring the intensity of the product (cut) RNA bands, with respect to DDX21_Fl_ activity. (E) Quantification of the G-quadruplex remodeling assay for the constructs with activity above background: Fl, ΔN, and the monomeric ΔN and Fl mutants. The graph on the right summarizes the results from three independent experiments (see also Figure S11). Error bars represent the standard deviation.

Using the acquired structural information, we sought to understand the influence of dimerization and each accessory domain in RNA binding and unwinding. The binding affinities of the DDX21 variants to ssRNA, dsRNA, and RNA G-quadruplex were measured using fluorescence polarization experiments (see Transparent Methods, Figures 3A, 3B, and 4A, and Table S1). DDX21_Fl_ binds RNA in the absence of ATP with the highest affinity for the RNA G-quadruplex (Q2 RNA) (2.6 nM Figure 4A, Table S1), whereas the affinity decreases to 30.7 and 10 nM, for ssRNA and dsRNA, respectively (Figures 3A and 3B). All constructs bind RNA with nanomolar affinity, but DDX21_Fl_ exhibits the strongest affinity, indicating that all the domains cooperate in stabilizing the complexes. Removal of the N-terminal 185-amino acid region has a slight effect on the binding to the ssRNA and the dsRNA (∼1.5-fold decrease) and a ∼3-fold decrease to the RNA G-quadruplex binding. These results indicate that this domain, most likely unstructured (see below), makes only a small contribution to RNA binding. On the other hand, deletion of the C-terminal basic tail significantly reduces the affinity by at least one order of magnitude for all substrates (DDX21_ΔNC_). The additional deletion of the GUCT domain in the DDX21_Core-DD_ construct slightly recovers this drop (by 1.5-fold), still remaining far below the DDX21_Fl_ binding affinity values. DDX21_Core_ is the weakest binder, whereas the monomeric DDX21 mutants bind RNA as tightly as the wild-type protein, emphasizing that the C-terminal basic tail is the key for RNA recognition, whereas dimerization seems not to be essential.

We next tested the dsRNA unwinding activity of the DDX21 mutants using an *in vitro* helicase assay (Figures 3C–3G and S8). Unwinding of the dsRNA substrate by DDX21 could not be observed directly by gel electrophoresis, as the single strands reannealed quickly (lane 4 in Figure 3C). Strand separation was observed only when boiling the samples at 95°C (lane 1 in Figure 3C). To observe the unwinding reaction, RNase T1 was used to cleave the resulting single RNA strands at G residues while the unwinding reaction was taking place (lane 5 in Figure 3C). Importantly, RNase T1 cleavage depends on the presence of both DDX21 and ATP (lane 5 versus lanes 4 and 6 in Figure 3C) demonstrating that DDX21 uses ATP hydrolysis for dsRNA unwinding. The helicase activity of the DDX21 mutants was compared with DDX21_Fl_ activity using a time course experiment (Figures 3D–3F). DDX21_ΔN_, without the N-terminal unstructured region, has ∼87% DDX21_Fl_ activity, confirming that this region is not necessary for RNA unwinding (Figure 3D). However, as shown in Figure 3E, deletion of the C-terminal basic tail reduces the activity to ∼15% of DDX21_Fl_. Additional removal of the GUCT domain does not decrease the activity further. DDX21_Core_ has almost undetectable activity under the same conditions. Interestingly, the monomeric DDX21 mutants, with intact GUCT domains and basic tails, have only ∼50% of the dimeric protein activity (Figure 3F), indicating that an intact DD is crucial for maintaining full dsRNA unwinding activity.

### DDX21 Requires Dimerization to Remodel RNA G-Quadruplexes

G-quadruplexes are non-canonical nucleic acid secondary structures found in sequences rich in guanine residues (Agarwala et al., 2015). Despite the importance of RNA G-quadruplexes homeostasis in cells, the understanding of the mechanisms underlying G-quadruplex processing by helicases is still poor. As DDX21 has the highest affinity to this RNA structure (Figures 3A, 3B and 4A), we investigated the implications of our structural model in the context of RNA G-quadruplex binding and remodeling. FP RNA-binding experiments show that ionic strength greatly affects the binding affinity of all DDX21 variants to such structures (Figure S9 and Table S2). The flexibility of DDX21 may allow modulation of the accessibility of charged patches on its surface to bind certain protein or RNA partners. The C-terminal basic tail is essential for the recognition of this RNA structure (Figure 4A), as shown by previous studies (McRae et al., 2017, 2018). Further deletion of the GUCT domain strengthens the interaction to a moderate extent. The GUCT domain thus seems to contribute little to RNA binding and remodeling by itself, but may support protein-protein interactions and the formation of an RNA binding unit together with the C-terminal basic tail (see next section). Such a role in protein-protein interactions for the GUCT domain was proposed for DDX50, by comparison of its pI with that of the rest of the protein and by observation of its electrostatic potential distribution (Ohnishi et al., 2009) (Figures S2 and S7). Therefore, although sharing a similar structure as other RRMs of bacterial enzymes, the GUCT domain of DDX21 might not share their RNA-binding modes.

Interestingly, the specificity for the RNA G-quadruplex is already present in the DDX21_Core-DD_ and DDX21_Core_ constructs, which bind this substrate with an order of magnitude higher affinity than all other substrates (Figures 3A, 3B and 4A). The aforementioned disruption of the dimerization interface does not affect the binding of DDX21 to RNA G-quadruplexes (Figure 4A), so we sought to determine whether oligomerization influences their remodeling.

DDX21 has been shown to remodel a G-rich RNA sequence from one G-quadruplex conformation to another (McRae et al., 2017). Circular dichroism spectroscopy and dynamic light scattering were used to assess the formation of the expected parallel G-quadruplex structure by the 5′Fluorescein amidite (FAM) Q2 RNA (Figure S10), which was maintained even in the presence of DDX21_Fl_ (Figure 4B). To monitor the remodeling activity of DDX21, we used a similar approach as before, using RNase T1 (Figures 4C–4E and S11). In this case, RNase T1 cuts the substrate in the absence of DDX21, with the presence of the helicase accentuating this activity, showing that DDX21 changes the conformation of this RNA structure increasing the accessibility of the G bases. As for dsRNA unwinding, DDX21_Fl_ has the highest activity, whereas only DDX21_ΔN_ and the monomeric mutants have activity above background. All the other truncation mutants are compromised (Figures 4C–4E). As opposed to dsRNA unwinding, G-quadruplex remodeling is not dependent on ATP (Figure 4C).

Taken together the data indicate that DDX21 uses its C-terminal accessory domains and dimerization to unwind dsRNA and specifically recognize and remodel RNA G-quadruplex structures.

### DDX21 Domain Architecture and Conformational Flexibility

Owing to the flexible nature of helicases, the combination of solution scattering (SAXS) data and flexible fitting of individual domain structures is a powerful tool to understand the conformation and the structure-function relationship in these multi-domain systems (Huen et al., 2017; Mallam et al., 2011; Rambo, 2015; Xu et al., 2017). We, therefore, used this method to explore the quaternary organization of DDX21 and study the spatial arrangement of the domains with respect to each other to better rationalize the role of dimerization for DDX21 activity.

**Figure 5:**
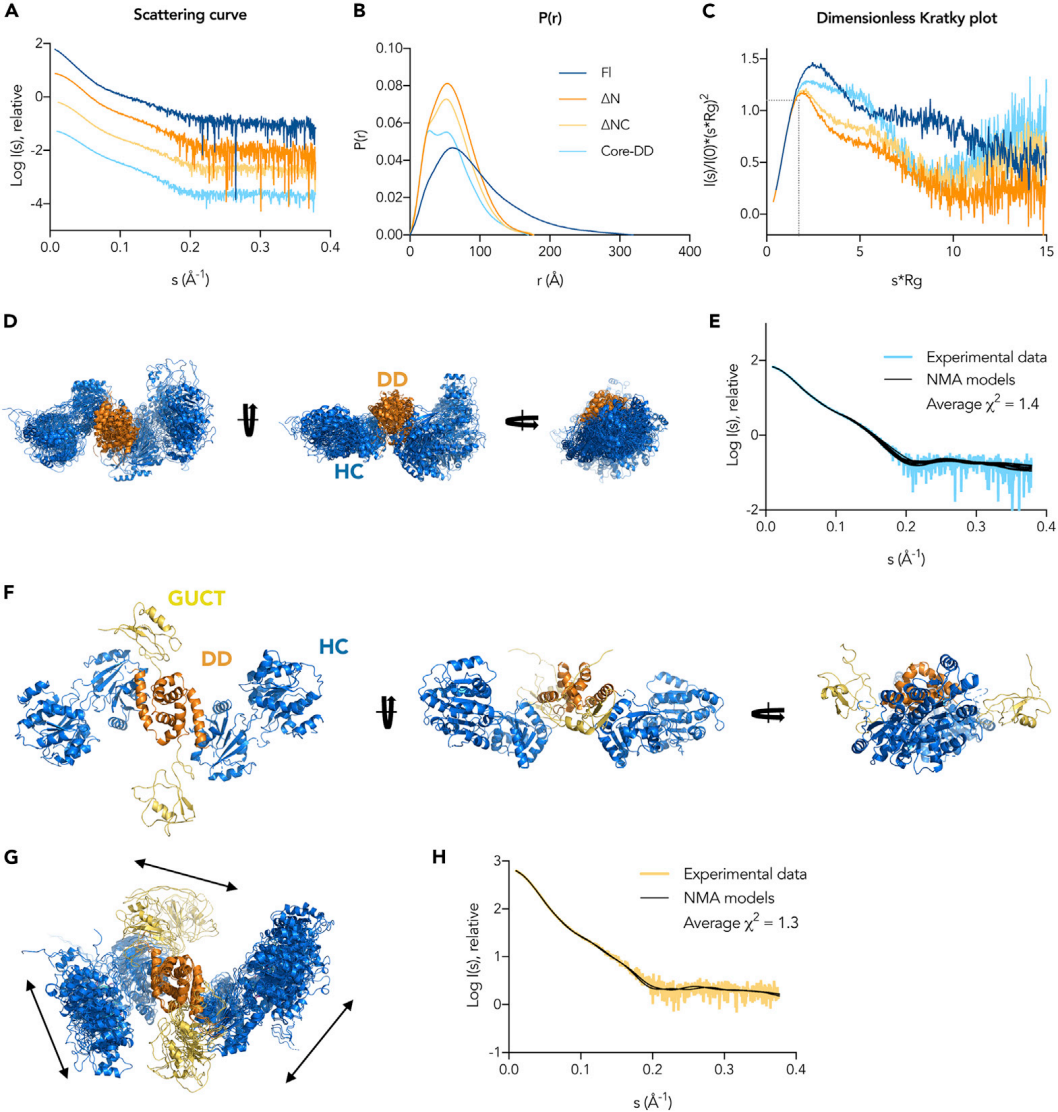
SAXS-Guided Modeling and DDX21 Conformational Flexibility (A) Experimental scattering curves generated from the SEC-SAXS traces shown in Figure S12A. (B) Pair distance distribution functions indicating the maximum dimension of the particles, D_max_. (C) Dimensionless Kratky plot shows the characteristic profile for flexible multi-domain proteins (see also Figures S12 and S13). (D) The ensemble of the outcome structures from the NOLB NMA for DDX21_Core-DD_ displays a linear arrangement of the HC and DD domains (see also Figure S14). (E) Comparison between the calculated scattering curves from the ensemble models (black) and the DDX21_Core-DD_ experimental scattering curve (light blue), giving an excellent fit with χ^2^ of 1.4. (F) Representative DDX21_ΔNC_ model of the ensembles created by flexible fitting, depicting the flatness of apo DDX21_ΔNC_ in solution. (G) Ensemble of the outcome structures from the NOLB NMA for DDX21_ΔNC_. The arrows indicate the domain movements after aligning the conformers by the DD. The HC domains are shown in blue, the DD in orange, and the GUCT domains in yellow. (H) Comparison between the calculated scattering curves from the ensemble models (black) and the DDX21_ΔNC_ experimental scattering curve (yellow), giving an excellent fit with χ^2^ of 1.3 (see also Figures S15–S17).

SAXS was performed coupled to size exclusion chromatography (SEC-SAXS) to avoid aggregation effects (Figure 5 and S12, the relevant parameters in Table S3). The Guinier analysis of the scattering curves (Figure S13) shows good linearity indicating neither aggregation nor polydispersity effects. According to SAXS molecular weights estimates, all the constructs (except the ΔN monomer mutant) are dimers, as expected (Table S3). The Guinier approximation gives an estimated radius of gyration (R_g_) of 70 Å for Fl, 48 Å for ΔN, 46 Å for ΔNC, and 44 Å for Core-DD. The large difference between the R_g_ value of the DDX21_Fl_ and the rest of the constructs suggests the presence of a core globular structure and an elongated flexible N terminus. This is apparent when comparing the pair distance distribution functions P(r) and the maximum dimensions (D_max_) for the DDX21_Fl_ and the variants in Figure 5B and Table S3. DDX21_Fl_ shows a profile characteristic of elongated structures with a very large D_max_ of 319 Å and a main peak at a much shorter radius (dark blue in Figure 5B). When the N-terminal 185 amino acids are removed, the P(r) function resembles that of a globular structure with a much smaller D_max_ of about 177 Å (orange in Figure 5B). In addition, the dimensionless Kratky plot (Figure 5C) contains features typical of multi-domain proteins with flexible linkers for all constructs, as expected, but indicates a larger flexibility for DDX21_Fl_ (dark blue). As a reference, compact, globular molecules show a peak at the Guinier-Kratky point (1.1, √3) where the dashed lines in Figure 5C intercept (Receveur-Brechot and Durand, 2012).

Compared with the compact globular shape previously described for the monomeric DDX21_Core_ (Chen et al., 2020), DDX21_Core-DD_ uncovers a flexible, elongated, and bimodal structure (as seen by the two peaks in the P(r) function in Figure 5B light blue), fitting to a dimer of two HC domains (Figures 5D and S14). In solution, DDX21_Core-DD_ does not form the “inverted V-shaped” structure as seen, for example, in the CshA crystal structure (Huen et al., 2017) (Figures S14A and S14B), but a more linear arrangement with the HC domains extending away from the DD (Figures 5D and S14C and S14D). Interestingly, the dimensionless Kratky plot in Figure 5C suggests that DDX21_Core-DD_ is less compact than DDX21_ΔNC_ and DDX21_ΔN_. Therefore, the SAXS data suggest that the presence of the GUCT domain makes the protein more compact. More importantly, DDX21_ΔN_, which contains the C-terminal basic tails with the F/PRGQR repeats, is still very compact, suggesting that the C-terminal tail provides structure. The basic tail expressed and purified alone in solution is soluble and unfolded (Figure S15). However, the R_g_ and D_max_ values for the DDX21_ΔNC_ and DDX21_ΔN_ constructs are very similar, suggesting that the basic tail may pack against the GUCT domain, and perhaps, contributes to the higher thermal stability of DDX21_ΔN_ with respect to DDX21_ΔNC_ (T_m_ (ΔN) = 62°C versus T_m_ (ΔNC) = 38°C, Figure S5B). In line with this hypothesis, inspection of the GUCT domain electrostatic surface clearly shows an exposed acidic surface (Figure S2C) that could be binding the basic tail.

We have reconstructed a complete model for DDX21_Fl_ without the unstructured N- and C- terminal tails, using the SAXS data to guide the modeling and analyze its flexibility (Figure 5E). We compared two approaches that can deal with multi-domain flexible systems in solution: the ensemble optimization method (EOM) (Bernadó et al., 2007; Tria et al., 2015) and the nonlinear rigid block normal-mode analysis method (NOLB NMA) (Hoffmann and Grudinin, 2017). EOM creates an ensemble of structures that together represent the scattering macromolecule (Figures S16). For the NOLB NMA procedure, DDX21_ΔNC_ models were modified to fit the SAXS experimental curve by efficiently exploring the configurations of the flexible linkers and rigid domains (see Transparent Methods and Figures 5E, 5F, and S17). Both EOM and NOLB NMA generate flexible conformational ensembles where the HC domains can be found in a range of open conformations characteristic of apo helicases. The two protomers are linked by the small helical DD, which becomes the only point of contact between them (Figure 5). From this central point, the GUCT domains also extend out in the same plane as the HC domains. The variations within the ensembles (depicted by arrows in Figures 5G and S17) result from the opening and closing of the HC and the movements of the GUCT domains with respect to the DD, highlighting the origin of flexibility. This structural organization places the DD at the center of the protein dimer, acting as a structural linker between the HC domains and the GUCT-basic tail units, coordinating their joined activities onto the RNA substrates, explaining why maintaining the dimer interface is essential for enzymatic activity.

## Discussion

We identify human DDX21 as a dimer and suggest a model of its quaternary structure relating these findings to its RNA-binding and unwinding activities. Using integrative modeling that combines biochemistry experiments, homology modeling, and SAXS, we found that the human DDX21 helicase is a homodimer, in which residues 570–620 form a hydrophobic dimerization domain similar to those found in other bacterial DEAD-box helicases. Sequence alignments and structural comparison using the Dali server (Holm, 2019) were unable to identify other human DEAD-box helicases containing an analogous dimerization domain. Consequently, this is a unique characteristic of the two human paralogs DDX21 and DDX50.

DDX21 can unwind dsRNA substrates with both 3' (this work) and 5' single-stranded overhangs (Valdez et al., 1997) in an ATP-dependent manner. We propose that DDX21 uses dimerization and the basic C-termini to recognize and unwind dsRNA substrates. Dimerization on a dsRNA substrate has only been observed in the crystal structure of the truncated HC of the DEAD-box helicase DDX3X in the pre-unwound state (Song and Ji, 2019). As observed for DDX3X and also the homologous bacterial CsdA (Xu et al., 2017), DDX21 dimerization allows for cooperativity and enhances the unwinding activity of duplexes. However, as opposed to DDX3X HC that binds dsRNA with a 2:1 protein:RNA stoichiometry, full-length DDX21 is able to form a complex with 2:2 stoichiometry (as seen for the R-loop substrate, Figure 2). Within the DDX21 dimer, there are two HCs and two GUCT-basic tail units that can bind two substrates simultaneously or perhaps also work synergistically to unwind or remodel one substrate RNA. The different stoichiometries may depend on the nature (shape and length) of the substrate and may have important functions in the cell.

DDX21_Fl_ binds RNA G-quadruplex with the highest affinity, when compared with other RNA structures, and this preference already exists in the DDX21_Core-DD_ and DDX21_Core_ constructs. Interestingly, the bacterial DEAD-box helicase CsdA also shows the same behavior, given that the DD-Core dimer of CsdA has specificity for the G-rich substrates over other RNA structures tested (Huen et al., 2017). As these interactions are ionic strength dependent, this preference for the G-quadruplex disk-like structures might be determined by the electrostatic surface charge distribution of the HC dimer. The accessory domains will contribute to enhancing the affinity of the dimer, modulating the electrostatic effects that govern RNA binding, as suggested here for the GUCT-basic tail unit of DDX21. As opposed to dsRNA unwinding, RNA G-quadruplex remodeling does not require ATP, indicating that the protein is using a different mechanism than that of canonical DEAD-box helicases. Still, an intact dimer interface is essential for maintaining this activity. *In vitro* experiments described by McRae et al. (2017, 2018) complement these findings as they show that the C-terminal domain of DDX21 can remodel G-quadruplexes in an ATP-independent manner. Their experiments used a construct covering the DD-GUCT and basic tail. In our experiments, we have separated the contributions of the C-terminal basic tail from the GUCT and dimerization domains, which allows us to show that dimerization in DDX21 is important also for its ATP-independent G-quadruplex remodeling function.

The combination of dimerization and C-terminal accessory domains seems to be a recurring feature within the DEAD-box family (as seen in the helicases Hera, CsdA, DDX21, and DDX50). This indicates that oligomerization cannot be overlooked when studying the mechanism of action of this helicase family. Further evidence on the role of oligomerization comes from the association of other helicases in cells (DDX5 and DDX17, Ogilvie et al., 2003) or *in vitro* (human DDX3X and its yeast ortholog Ded1p, Putnam et al., 2015; Sharma et al., 2017; Song and Ji, 2019). DDX3X and Ded1p are reported to function as trimers based on the estimation of their rate constants in unwinding reactions that show a sigmoidal dependence on helicase concentration (Sharma et al., 2017; Yang and Jankowsky, 2006). Both of them contain a low-complexity region at their C termini, which turned out to be essential for oligomerization. Human DDX21 differs from these eukaryotic enzymes as, based on the present results, it functions as a dimer, containing a dimerization domain that is shared with bacterial helicase homologs. Therefore, eukaryotic helicases seem to have the capacity to define and control their activity using different accessory domains able to promote specific oligomeric states.

DEAD-box RNA helicases have indispensable roles in cell homeostasis; however, our understanding of their RNA recognition and function in the cell at the molecular and structural levels is sparse primarily because these enzymes are challenging to characterize structurally given their flexible and multi-domain nature. As further studies are necessary for understanding the implications of oligomerization on the catalytic mechanism, as well as on the recognition of binding partners (in the case of DDX21 these could be phosphatases, viral proteins, or the 7SK RNP complex, for instance, Calo et al., 2015; Chen et al., 2014; De Wever et al., 2012), these findings will contribute to shed light on the molecular mechanisms of DEAD-box helicases in the cell and their potential relevance as therapeutic targets.

### Limitations of the Study

The fact that the activity assays presented here were carried out using purified protein and *in vitro* conditions might be a limitation of the work, as we are not studying the enzyme in the cellular environment in conjunction with protein or nucleic acid partners. In addition, the models presented are at medium-low resolution given the experimental data available and have to be interpreted at this resolution level.

### Resource Availability

#### Lead Contact

Further information and requests for resources and reagents should be directed to and will be fulfilled by the Lead Contact, Matteo Dal Peraro (matteo.dalperaro@epfl.ch).

#### Materials Availability

All unique/stable reagents generated in this study are available from the Lead Contact without restriction.

#### Data and Code Availability

SAXS data and models were deposited in the Small Angle Scattering Biological Data Bank SASBDB with accession codes SASDGU9, SASDGV9, SASDGW9, SASDGX9, and SASDGY9.

## Methods

All methods can be found in the accompanying Transparent Methods supplemental file.

## Supplemental Information

Document S1. Figures S1–S17, and Tables S1–S3, and Transparent Methods
