# Supplementary material for: The Human RNA Helicase DDX21 Presents a Dimerization Interface Necessary for Helicase Activity
Source: iScience. 2020 Nov 18;23(12):101811. doi: 10.1016/j.isci.2020.101811 (PMC7721647; doi:10.1016/j.isci.2020.101811)
Supplement: Document S1. Figures S1–S17, and Tables S1–S3, and Transparent Methods [file mmc1.pdf]

iScience, Volume *23*

Supplemental Information

The Human RNA Helicase DDX21 Presents a Dimerization Interface Necessary for Helicase Activity

Maria J. Marcaida, Annamaria Kauzlaric, Alice Duperrex, Jenny Sülzle, Martin C. Moncrieffe, Damilola Adebajo, Suliana Manley, Didier Trono, and Matteo Dal Peraro

Content

- Supplemental Figures S1-S17
- Supplemental Tables S1-S3
- Transparent Methods
- Supplemental References

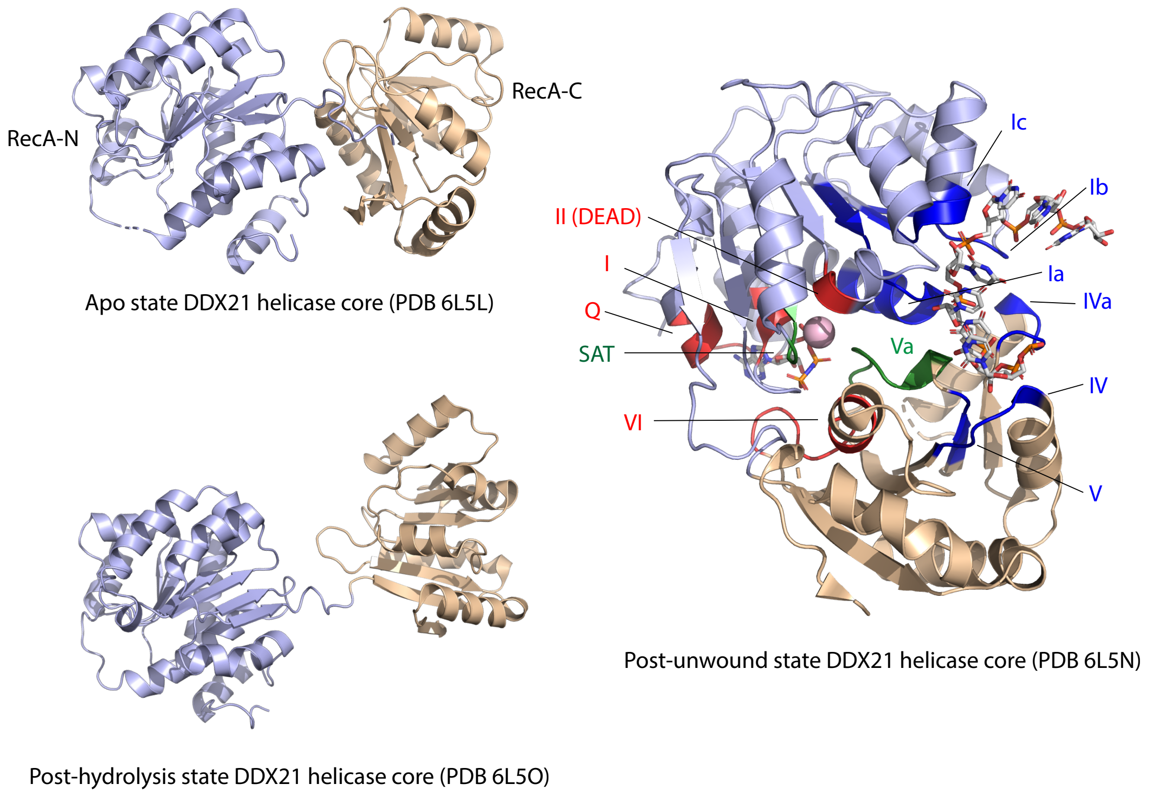

Figure S1. Crystal structure of the DDX21 HC (Related to Figure 1A). Cartoon representation of the DDX21 helicase core domain in three conformations of the two RecA-like domains (RecA-N in light blue and RecA-C in wheat colour) (Chen et al., 2020). On the left, two crystal structures are shown as examples of the various open conformations (apo state PDB 6L5L and post-hydrolysis state PDB 6L5O) to highlight the range of movement allowed by the flexible linker. On the right, the crystal structure of DDX21 HC in complex with ssRNA, magnesium (pink sphere) and an ATP analogue, AMPPNP (PDB 6L5N) represents the post-unwound (closed) conformation. The characteristic motifs of DEAD-box helicases are indicated as follows: motifs interacting with ATP in red, motifs interacting with RNA in blue, and motifs involved in intramolecular interactions in green (Linder and Jankowsky, 2011).

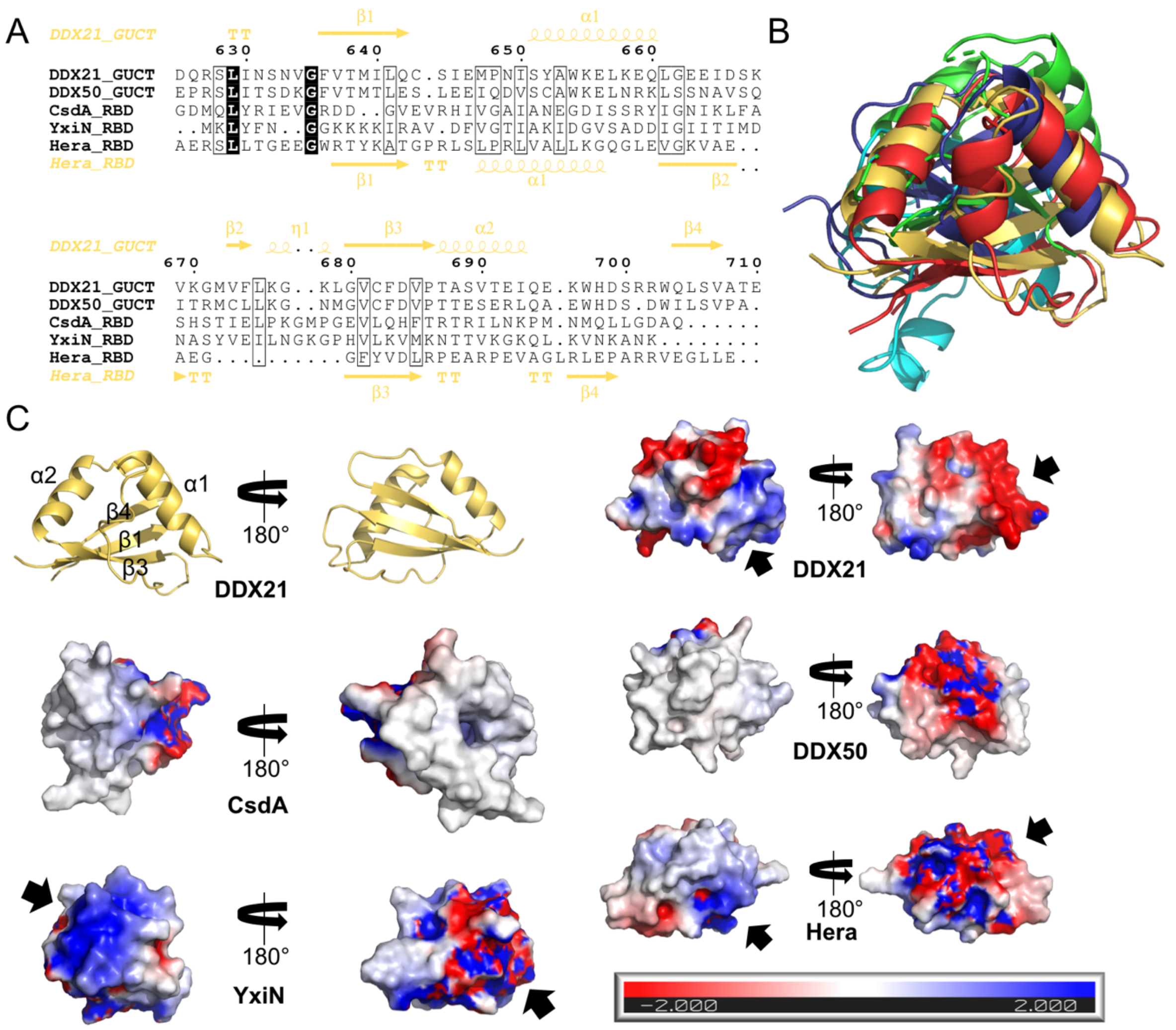

Figure S2. Analysis of the GUCT domain (Related to Figure 1A). A. Sequence alignment of five RNA binding domains (RBDs) or RNA recognition motifs (RRMs) from DEAD-box helicases shows very little sequence conservation. UniProt codes: DDX21: Q9NR30; DDX50: Q9BQ39; CsdA: P0A9P6; YxiN: P42305 and Hera: Q72GF3. Strictly conserved residues are highlighted in black, and residues with a global similarity score above 0.7 (from 0 to 1) are boxed. The secondary structure of the DDX21 GUCT domain is depicted on top, coloured in yellow. The sequence identity between DDX21 GUCT and the RRMs from Hera, CsdA and YxiN is ~30% and ~50% with the GUCT domain of DDX50. **B**. Superposition of the five RBD structures shows an overall fold conservation but Hera, YxiN and CsdA RBDs seem to have different RNA binding modes (Hardin et al., 2010; Ohnishi et al., 2009; Steimer et al., 2013; Xu et al., 2017). Some of the secondary structure elements are more conserved than others, namely α1-helix and strands β1, β3 and β4. The RBD from Hera is shown in blue, from CsdA in cyan, from DDX50 in red, from YxiN in green and from DDX21 in yellow. **C**. Cartoon representation of the GUCT domain of DDX21 showing the position of the secondary structure elements and comparison of the electrostatic potential surfaces of the different RBDs (in the same views as in the cartoon representation). The electrostatic potential distribution was calculated using APBS Electrostatics and the negative and positive potential values (from -2 to 2 kT/e) are shown in red and blue, respectively. RBDs from CsdA and DDX50 do not display obvious charged patches on their surface, but a large hydrophobic area. On the other hand, Hera, Yxin and DDX21 present positively charged regions and DDX21 shows a very large negatively charged patch (all indicated by black arrows).

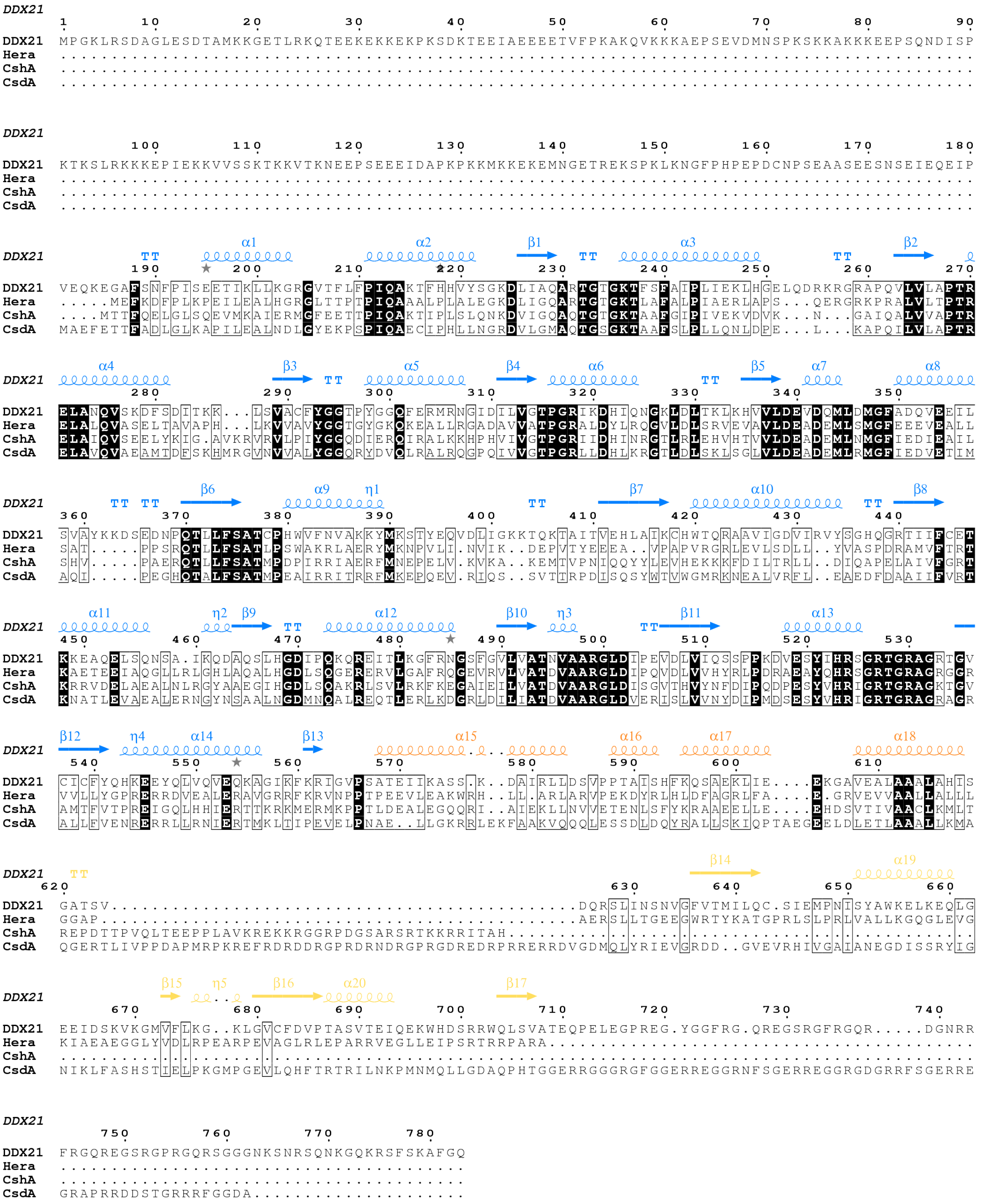

Figure S3. Sequence alignment (Related to Figure 1). Sequence alignment of DDX21 and the three bacterial dimeric DEAD-box helicases used as templates for homology modelling: Hera (Heat resistant RNA-dependent ATPase) from *Thermus thermophilus* (Klostermeier, 2013); CshA (Cold shock helicase A) from the Gram-positive bacteria *Geobacillus stearothermophilus* (Huen et al., 2017); and CsdA (Cold-shock DEAD-box protein A) from *Escherichia coli* (*E. coli*) (UniProt codes: DDX21: Q9NR30; Hera: Q72GF3; CshA: A0A0K2H973 and CsdA: P0A9P6). Strictly conserved residues are highlighted in black, and residues with a global similarity score above 0.7 (from 0 to 1) are boxed. The secondary structure of the DDX21 model is depicted on top, coloured as the domains in the main figures, namely, helicase core in blue, dimerization domain in orange and GUCT domain in yellow.

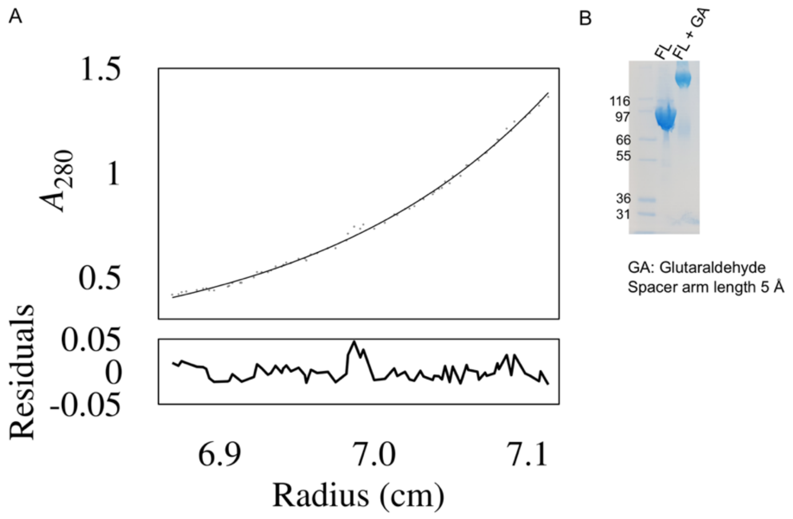

Figure S4. DDX21 is a homodimer (Related to Figure 1). A. Analytical ultracentrifugation equilibrium sedimentation experiment measures a molecular weight (Mw) for DDX21$_{Fl}$ of 154 kDa, agreeing with a dimer species (15% error as dimer Mw is 182 kDa). **B**. DDX21$_{Fl}$ was covalently crosslinked with glutaraldehyde, resulting in protein oligomers having molecular weights consistent with dimeric forms on SDS-PAGE.

A

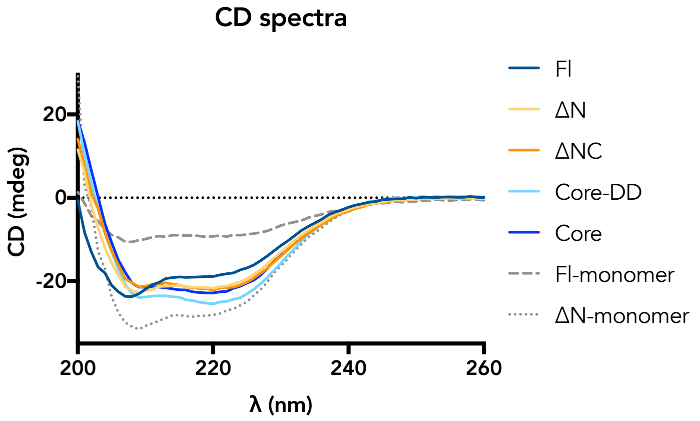

B

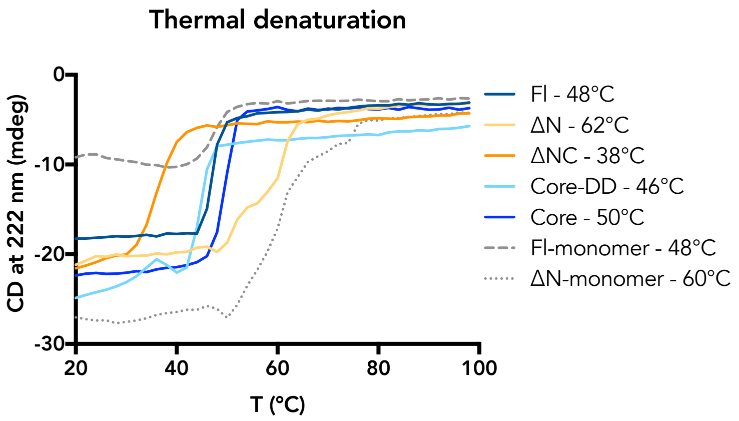

Figure S5. Folding and thermal stability of the DDX21 variants measured by circular dichroism (CD) (Related to Figure 1). A. CD spectra indicating that all the variants have secondary structure. **B**. Thermal denaturation curves monitored by measuring the CD signal at 222 nm across a temperature range from 20 to 98°C. The melting temperature T_m was calculated: T_m (Fl) = 48°C; T_m (ΔN) = 62°C; T_m (ΔNC) = 38°C; T_m (Core-DD) = 46°C; T_m (Core) = 50°C; T_m (Fl-monomer) = 48°C; T_m (ΔN-monomer) = 60°C. The T_m measured for $DDX21_{Fl}$ is the same as the one measured previously (McRae et al., 2017).

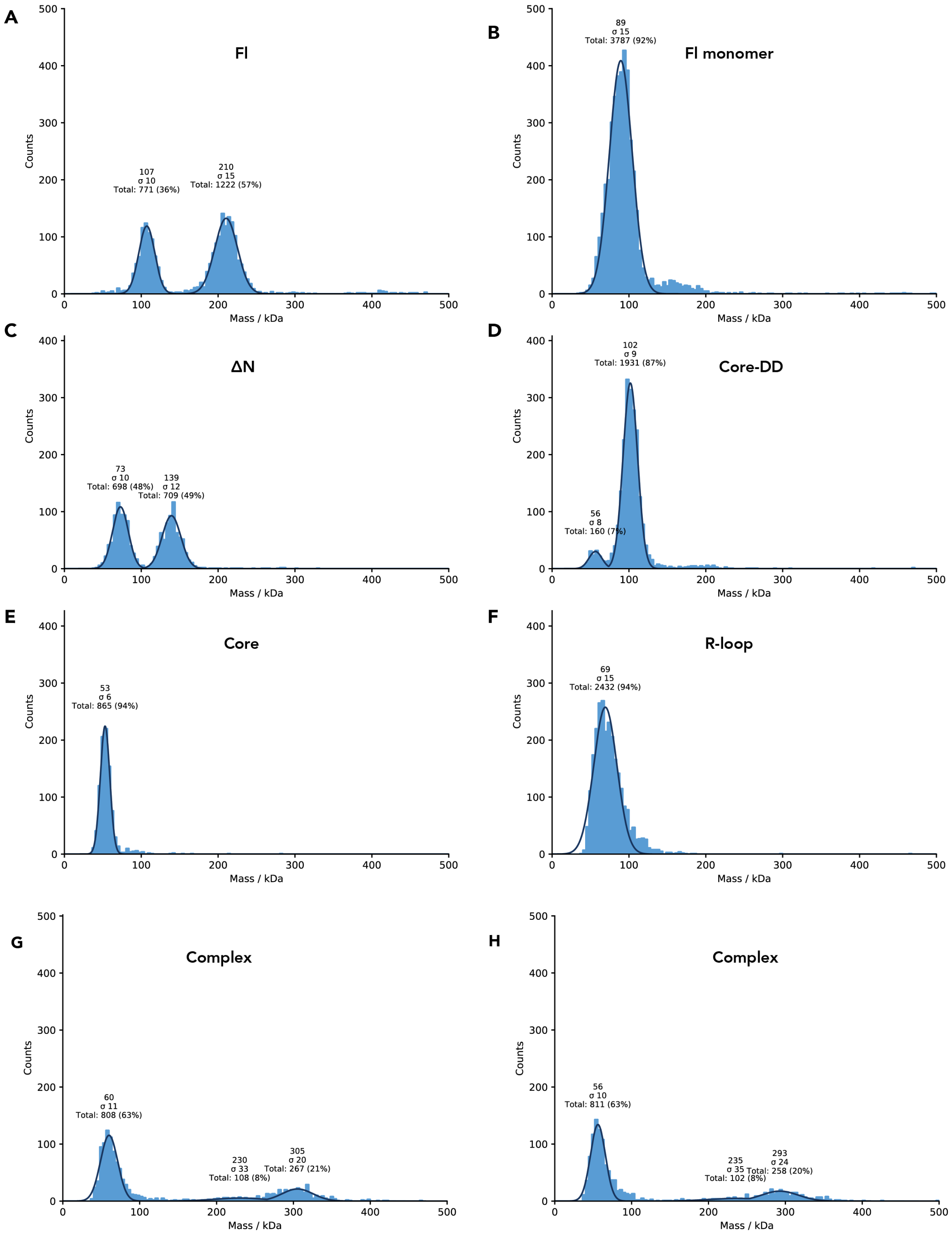

Figure S6. Replicas of the mass photometry measurements (Related to Figure 2). The samples were measured at the following concentrations: Fl at 30nM, Fl monomer mutant at 15nM; ΔN at 30nM, Core-DD at 15nM, Core at 15nM, R-loop at 15nM and the Fl-R-loop complex at 30nM.

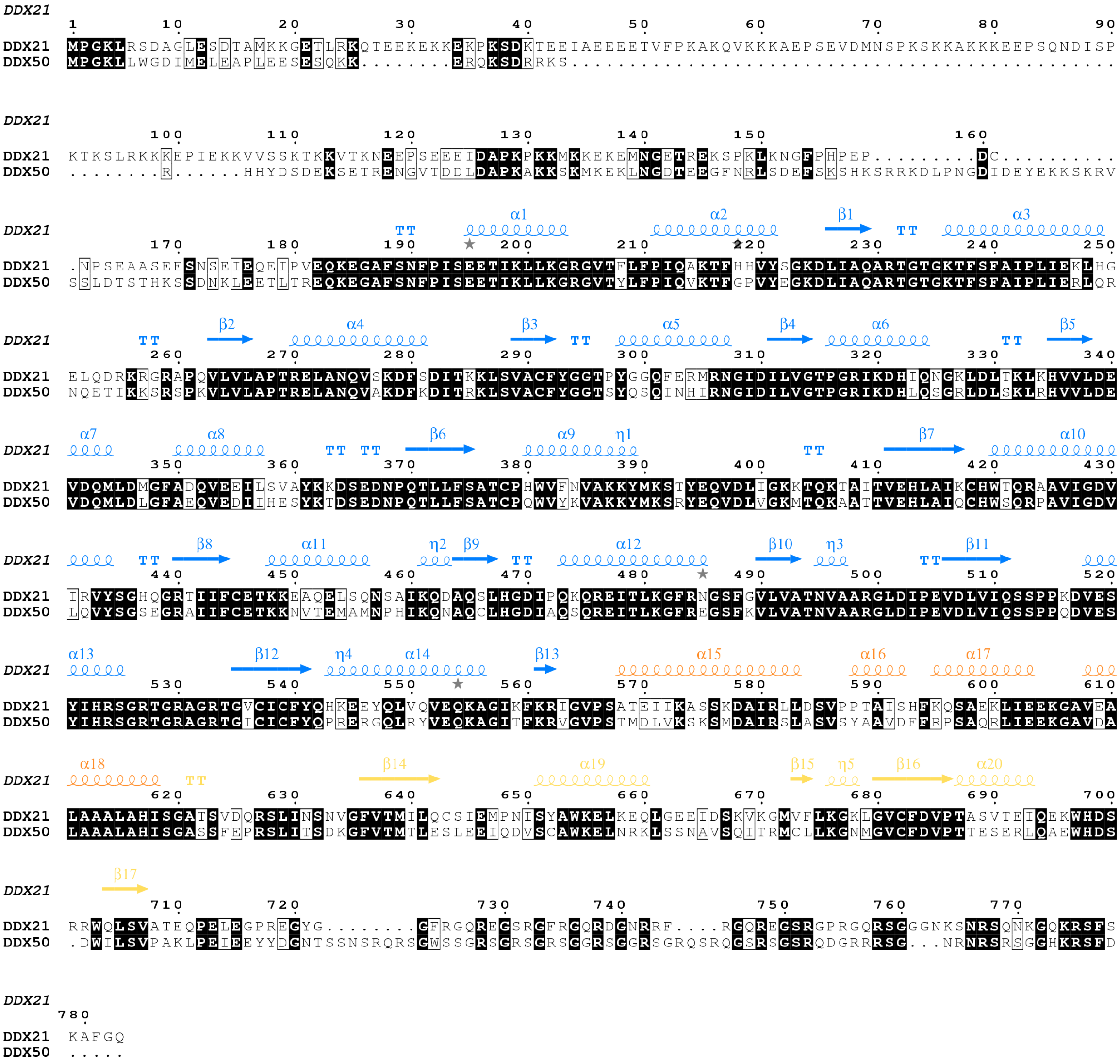

Figure S7. Sequence alignment of the two human paralogues, RH-II/Guα and RH-II/Guβ, i.e., DDX21 and DDX50 (Related to Figure 1). DDX50 localizes to nucleoli and nuclear speckles and may be involved in ribosomal RNA production and pre-mRNA splicing (Valdez et al., 2002a, 2002b). DDX21 and DDX50 share a similar domain architecture with an overall sequence identity of 55.6%. We suggest DDX50 may also form dimers. Another point worth highlighting is that the characteristic (F/P)RGQR repeats in the C-terminal tail of DDX21 are replaced by an arginine-serine-rich sequence in the C-terminal tail of DDX50 (Ohnishi et al., 2009; Valdez et al., 2002a). This may be the reason why DDX50 did not have RNA-folding activity (or misinterpreted G-quadruplexes helicase activity) *in vitro* (McRae et al., 2017; Valdez et al., 2002a). Colour coding and details of the alignment are as in **Figure S3.**

Fl, ∆N, ∆N monomer, ∆NC and Core-DD dsRNA unwinding assay 1:

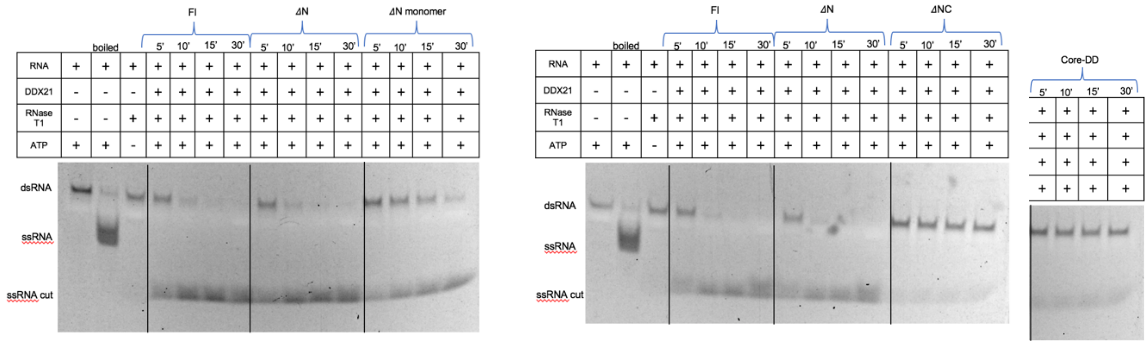

Fl, ∆N, ∆N monomer, ∆NC and Core-DD dsRNA unwinding assay 2:

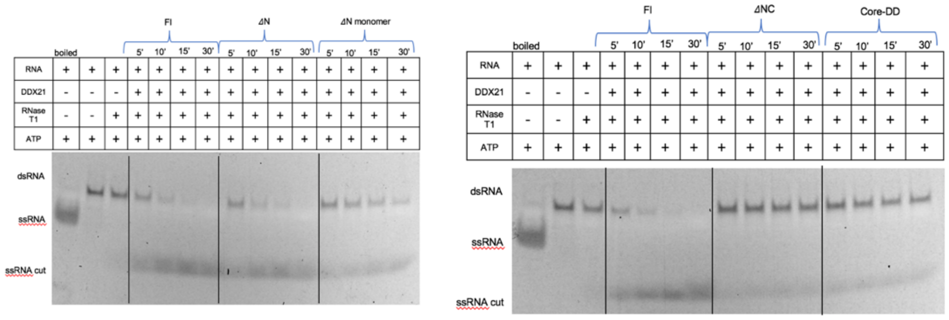

Fl, ∆N, ∆N monomer, ∆NC and Core-DD dsRNA unwinding assay 3:

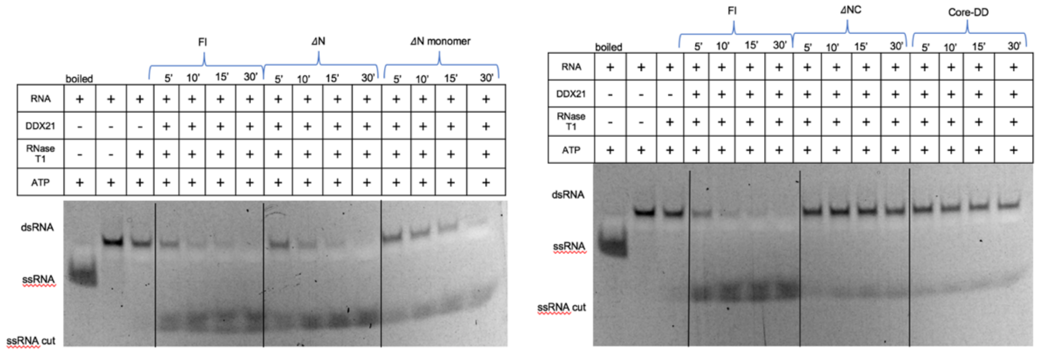

Fl monomer and Core dsRNA unwinding assays:

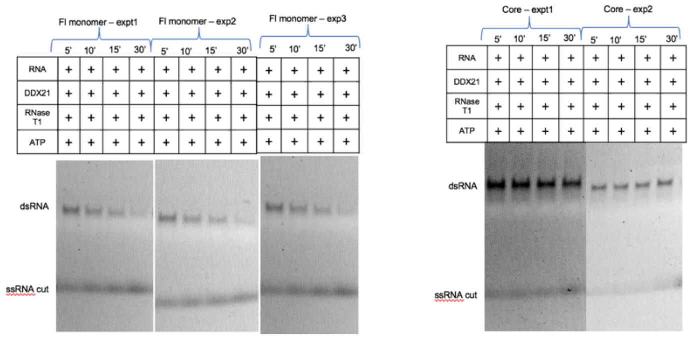

Figure S8. dsRNA helicase assays in triplicates (Related to Figure 3).

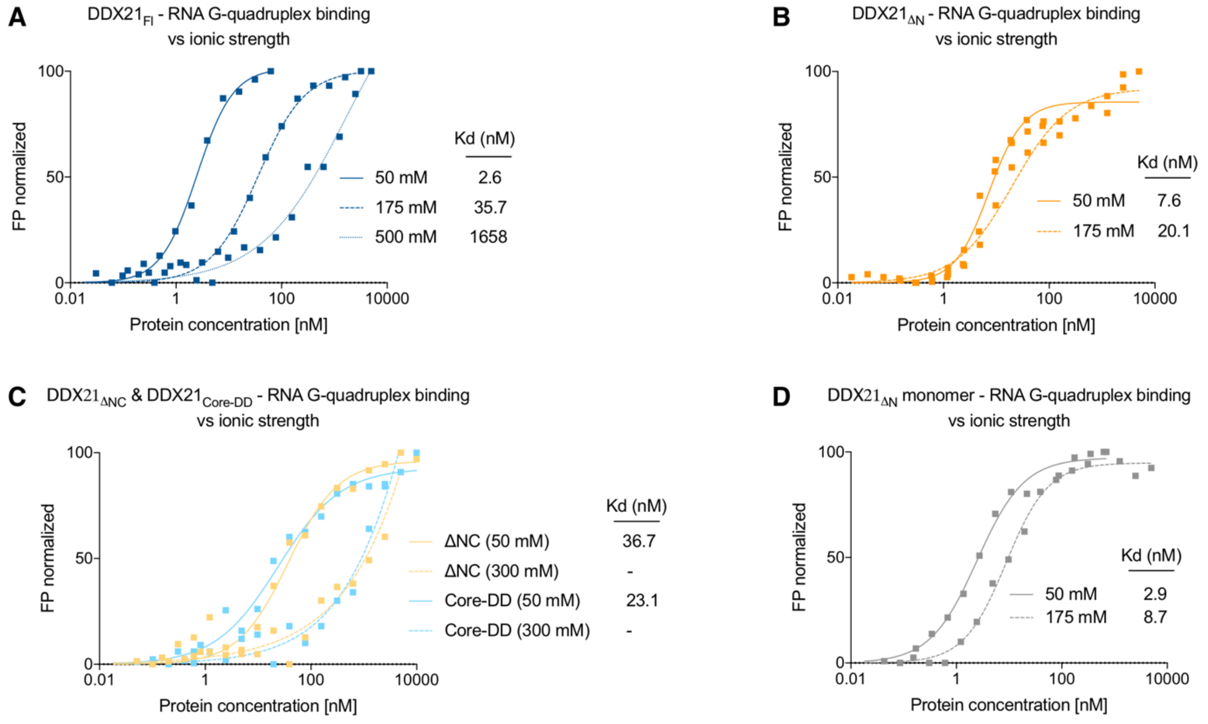

Figure S9. DDX21Fl - RNA G-quadruplex binding affinity versus ionic strength (Related to Figure 4). **A-D.** FP binding curves for the DDX21 variants and the RNA G-quadruplex under different salt concentrations. The $DDX21_{Fl}$ affinity for the RNA G-quadruplex decreases 14- and over 600-fold when the salt concentration increases from 50 mM to 175 mM and 500 mM, respectively (**Table S2**). This effect is also observed for all the mutants indicating that the recognition of the RNA is based on ionic interactions, as expected.

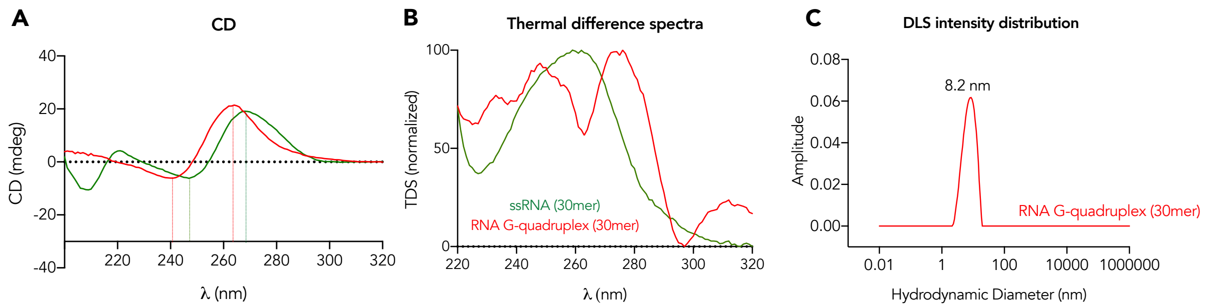

Figure S10. RNA G-quadruplex formation (Related to Figure 4B). A. CD spectra of the Q2 RNA G-quadruplex (30-mer- GUU GGG GCG GGC GUU GGG UUU GGG GGG ACG) used in the remodelling assays (in red) and a 30-mer ssRNA with sequence GCG UCG AUC CGA AAC UAU ACU UAA UUU UAA (in green) that does not form a G-quadruplex. The overlay clearly shows that the minima and maxima do not coincide. **B.** Thermal difference spectra of the two RNA molecules display the characteristic profiles of ssRNA and RNA G-quadruplexes (Mergny et al., 2005). **C.** Dynamic light scattering (DLS) was used to detect a globular structure with a hydrodynamic diameter of ~8.2 nm for the Q2 RNA G-quadruplex, in the same order of magnitude as that described by (Ariyo et al., 2015).

FI, FI monomer, ∆N and ∆N monomer RNA G-quadruplex remodelling assays

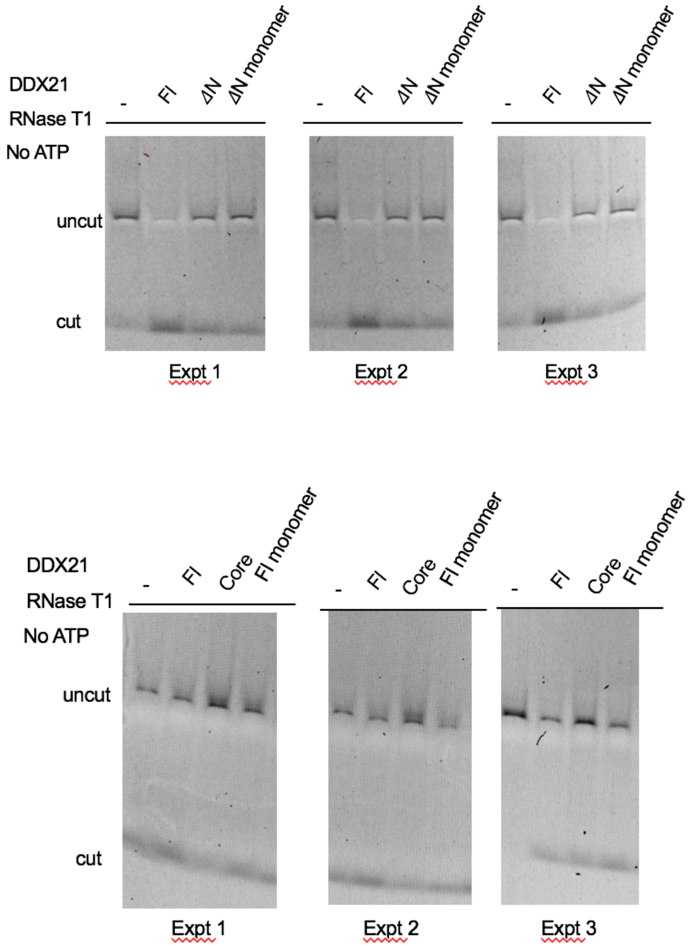

Figure S11. RNA G-quadruplex remodelling assays in triplicates (Related to Figure 4).

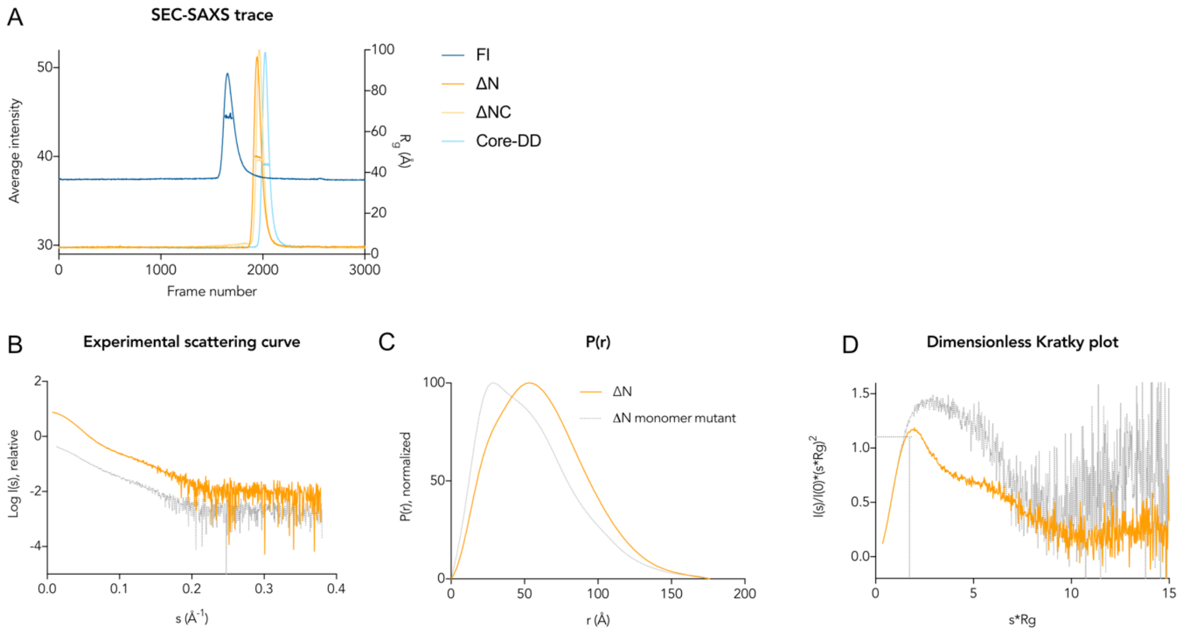

Figure S12. SEC-SAXS analysis of DDX21 (Related to Figure 5). **A**. SEC-SAXS elution profiles used to generate the data in **Figure 5**. The estimated R_g values for the peak regions are also shown. **B-D**. Comparison between DDX21$_{\Delta N}$ and its monomeric mutant (residues 611 to 616 (LAAALA) mutated to arginines) in orange and grey, respectively, using SAXS. **B**. Experimental scattering curve. **C**. Pair distance distribution functions indicating the D_{max} of both proteins is around 177 Å. **D**. Dimensionless Kratky plot shows the characteristic profile for multidomain proteins for ΔN and indicates more flexibility for the monomeric mutant.

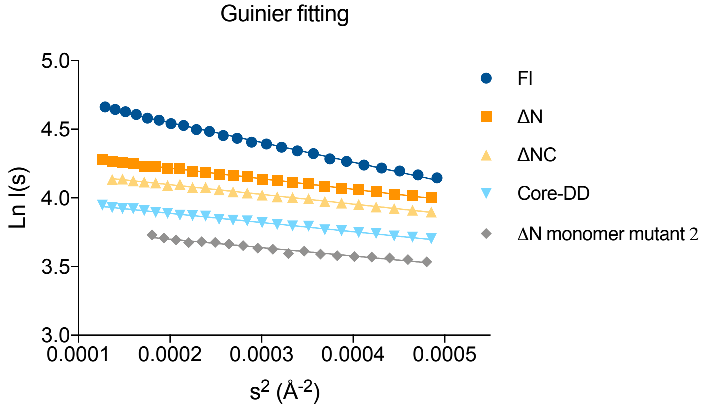

Figure S13. Guinier fitting of the SAXS scattering curves for the DDX21 variants shows no interparticle aggregation effects (Related to Figure 5).

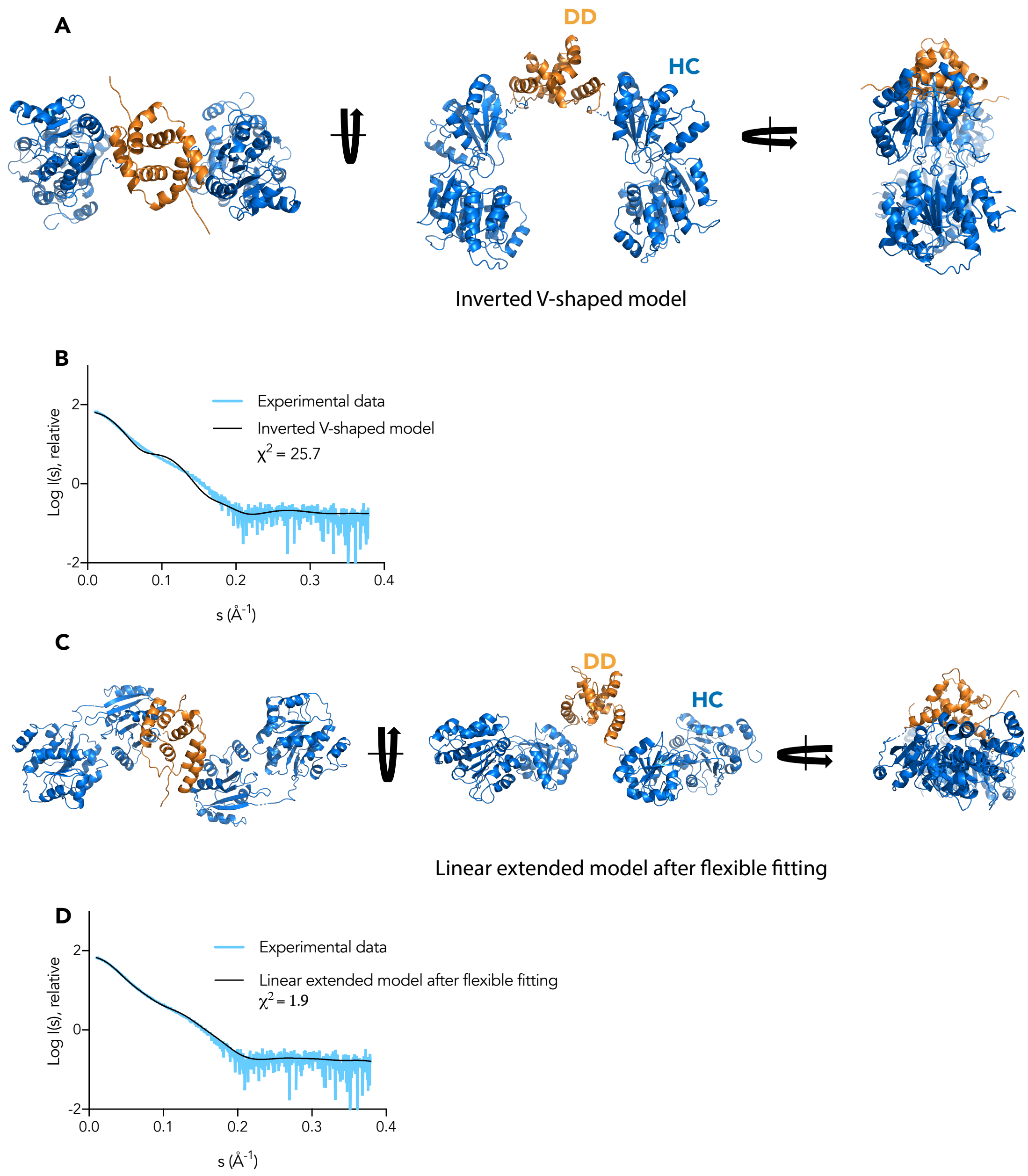

Figure S14. DDX21$_{\text{Core-DD}}$ conformation in solution (Related to Figure 5). A. Cartoon representation of the model of DDX21$_{\text{Core-DD}}$ based on the inverted V-shape conformation displayed by the CshA crystal structure (PDB ID 5IVL, (Huen et al., 2017)). **B.** The calculated scattering profile from the structure shown in **A** (black line) does not fit the experimental SAXS scattering curve (light blue line) (χ^2 = 25.7). Most likely, DDX21$_{\text{Core-DD}}$ adopts a more linear arrangement of its domains as shown in **C.**, whose calculated scattering profile fits much better to the experimental data (χ^2 = 1.9), shown in **D.**

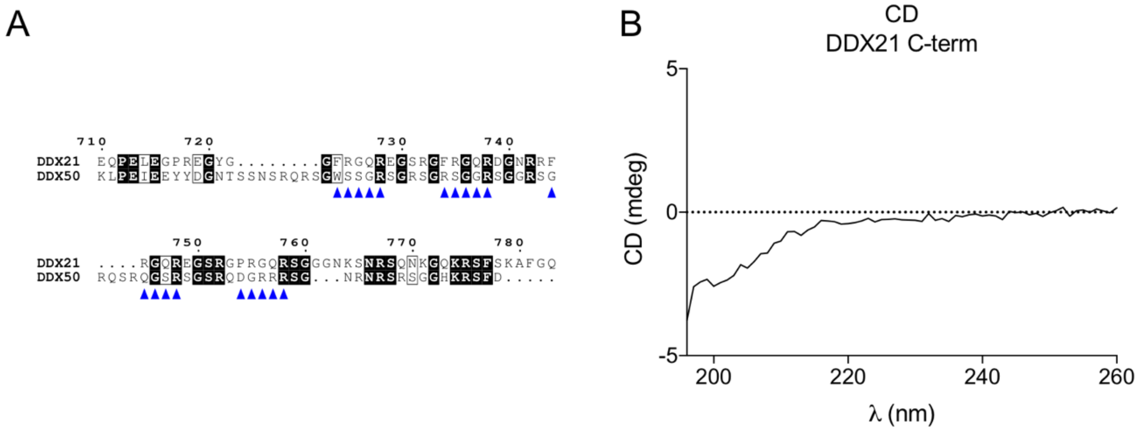

Figure S15. The C-terminal amino acids of DDX21 are unfolded (Related to Figure 5). **A**. C-terminal amino acids of DDX21 aligned to those from DDX50. The F/PRGQR repeats are highlighted by blue triangles. **B**. CD spectrum of the purified C-terminal domain alone shows it has no secondary structure, agreeing with predictions based on sequence alignments (JPred4 (Drozdetskiy et al., 2015)).

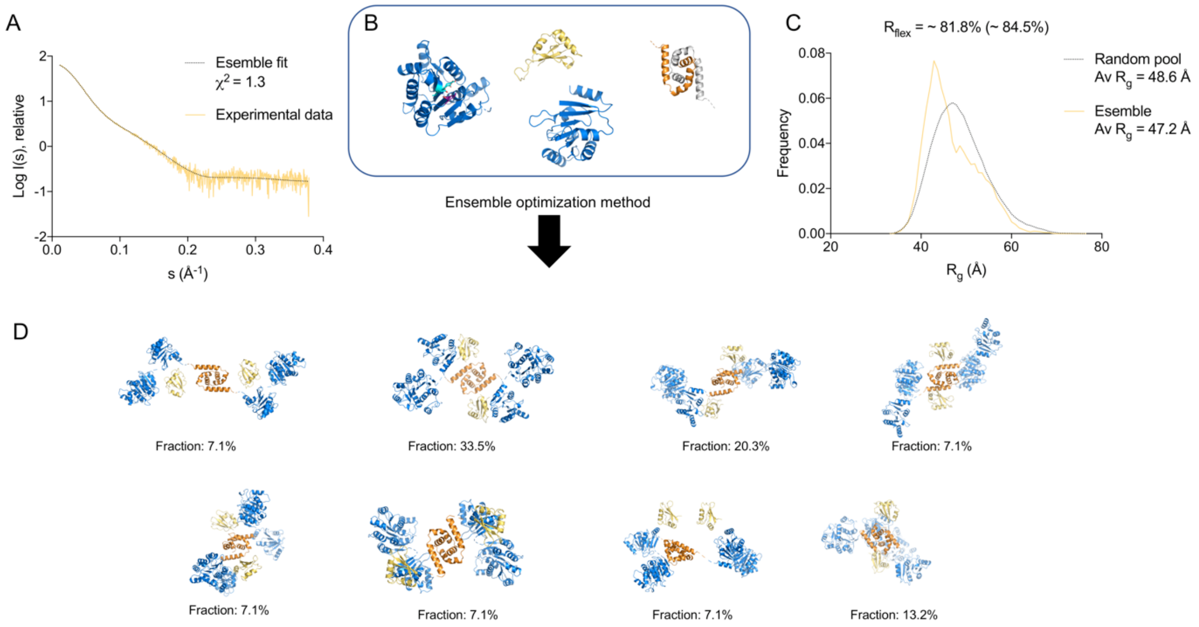

Figure S16. EOM analysis of DDX21$_{\Delta NC}$ (Related to Figure 5). A. Comparison between the calculated scattering curve from the whole EOM ensemble (black) and the DDX21$_{\Delta NC}$ experimental scattering curve (yellow), giving an excellent fit with χ^2 of 1.3. **B**. Input models for the globular domains. **C**. The R_g distribution for the random pool of models (dotted black) and for the ensemble of conformations that altogether give a good fit to the scattering intensity curve (yellow) are shown, with R_{flex} values of 84 and 81%, respectively. The distributions of the calculated R_g values are very close to those of the random pools and R_{flex} for the ensembles are slightly smaller to those from the random pools, indicating that the protein is highly flexible. **D**. Cartoon representation of the eight conformers in the EOM ensemble, showing the conserved flat structure. The frequency at which each conformer is present within the ensemble is shown as % fraction below each model. The HC is coloured in blue, the DD in orange and the GUCT domain in yellow.

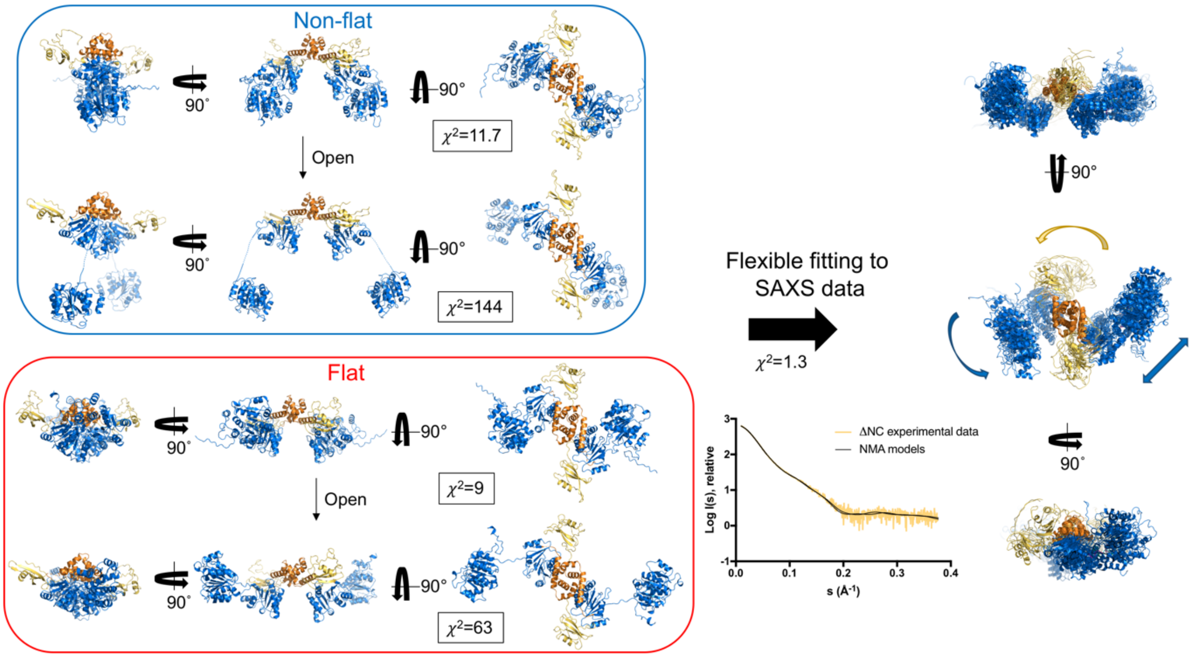

Figure S17. NOLB NMA of DDX21$_{\Delta NC}$ (Related to Figure 5). DDX21$_{\Delta NC}$ input models for the NOLB NMA procedure are shown in cartoon representation, grouped by the conformation of the helicase core (blue) with respect to the DD (orange): flat or not-flat ("inverted V-shaped"). The χ^2 values before the flexible fitting are indicated next to each model. The ensemble of resulting structures is shown on the right, where the arrows highlight the large movements of the domains. The final ensembles have a good fit to the experimental scattering curve with an average χ^2 value of 1.3.

Table S1. K_d values (nM) obtained from FP measurements (Related to Figures 3 and 4). The mean values from three independent measurements and their standard deviation were used to make the non-linear fit to the equation "Specific binding with Hill slope" in Prism, to calculate the K_d values and their uncertainties.

	Fl	ΔN	ΔNC	Core-DD	Core	Fl monomer	ΔN monomer
G-quadruplex	2.6±0.1	7.6 ±0.5	36.5±2.7	23.1±3.2	62.5 ± 4.9	9.8 ±0.9	2.9±0.3
ssRNA	30.7 ± 2.8	40.3±2.6	687.7±52.7	330.4±19.1	1525 ± 112.2	29.9 ± 2.7	24.9±1.7
dsRNA	10.2±0.4	15.5±0.8	217.4±10.4	143.2±6.1	353.3 ± 27.9	13.9 ± 2.5	121.8±19.3

Table S2. K_d values (nM) obtained from FP measurements using RNA G-quadruplex and different salt concentrations (Related to Figure 4). The mean values from three independent measurements and their standard deviation were used to make the non-linear fit to the equation "Specific binding with Hill slope" in Prism, to calculate the K_d values and their uncertainties.

	Fl	ΔN	ΔNC	Core-DD	ΔN monomer
50 mM*	2.6±0.1	7.6 ± 0.5	36.7±2.7	23.1±3.2	2.9±0.3
175/300 mM**	35.7±2.8	20.1±3.8	-	-	8.7±0.8
500 mM***	1658±1532	NM	NM	NM	NM

K_d values in nM. NM = Not measured; - = weak binding, K_d cannot be fit. *50 mM Tris pH 7.5, 50 mM KCl, 5 mM $MgCl_2$. **50mM Tris pH 7.5, 300 mM NaCl/175 mM KCl, 5 mM $MgCl_2$. ***25 mM Tris, 10 mM Hepes, pH 7.5, 25 mM KCl, 500 mM NaCl, 2.5 mM $MgCl_2$, 5% glycerol.

Table S3. SAXS parameters (Related to Figure 5).

	**Fl**	**ΔN**	**ΔNC**	**Core-DD**	**ΔN-monomer**
**SASPDB accession code**	SASDGU9	SASDGV9	SASDGW9	SASDGX9	SASDGY9
**Data collection**					
Instrument	ESRF BM29				
Beam size at sample (μm)	700x700				
Wavelength (Å)	0.992				
s range ($Å^{-1}$)	0.0025-0.500				
Detector	Pilatus 1M				
Detector distance (m)	2.867				
Exposure (s per image)	1				
Column	Superose 6 Increase 10/300				
Flow rate (ml/min)	0.5				
Sample volume (μl)	100				
Sample concentration (mg/ml)	9-12				
Temperature (K)	293				
**Structural parameters**					
R_g (Å) Guinier	70	48	46	44	41
R_g (Å) P(r)	76	49	47	46	44
D_{max} (Å)	319	176	177	169	177
Porod volume ($Å^3$)	681000	297000	238000	148000	111000
**Molecular mass determination**					
Theoretical MW (kDa)	90	68.7	60.6	49	69
MALLS MW (kDa)	197.7	143.2	120.5	91.4	68
DATPOROD MW (kDa)	400	174	140	87	65
DATVC MW (kDa)	227	156	122	88	68
DATMOW MW (kDa)	232	165	120	91	74
**Data analysis software**					
Data reduction	PRIMUS & ScÅtter				
Ab initio modelling	DAMMIF				
Homology modelling	Swiss Model				
Theoretical scattering curve calculation from models	Pepsi-SAXS				
3D graphics representation	Pymol/Chimera				

Transparent Methods

Protein expression and purification

The human DDX21 (Uniprot sequence Q9NR30, residues 1-783) expression plasmid carries the gene for the full-length protein in the pET-32 vector, with N-terminal Trx & Hexa-histidine tags, followed by a thrombin cleavage site. DDX21$_{\Delta N}$ (residues 186-783), DDX21$_{\Delta NC}$ (residues 186-710), DDX21$_{Core}$ (residues 186-564), DDX21$_{Fl}$ monomer (residues 1-783 with the 611LAAALA616 to 611RRRRRR616 mutation), DDX21$_{\Delta N}$ monomer (residues 186-783 with the 611LAAALA616 to 611RRRRRR616 mutation) and the C-terminal basic tail (residues 704-783) were cloned in the first multi-cloning site in the pET-DuetI vector, with a N-terminal Hexa-histidine tag followed by a TEV (Tobacco Etch Virus) cleavage site. DDX21$_{Core-DD}$ (residues 186-620) was cloned with an uncleavable C-terminal hexa-histidine tag in the pET-28b(+). The coding sequences for all the variants, except Fl, were optimized for *Escherichia coli* (*E. coli*) expression (Genscript). The plasmids were transformed in Rosetta (DE3) cells (Promega). Protein expression was induced by the addition of 0.1 mM isopropyl β-D-1-thiogalactopyranoside (IPTG) when the cells reached an optical density of 0.6 and subsequent growth over night at 20°C. Cell pellets were resuspended in lysis buffer (20 mM HEPES, pH 7.5, 500 mM NaCl, 10% (v/v) glycerol, 5 mM Imidazole and cOmplete™ Protease Inhibitor Cocktail (Roche)) and then lysed using sonication. Poly(ethyleneimine) solution (0.1% v/v) was added to the resulting suspensions which were then centrifuged (13000 rpm for 35 min at 4°C) and the supernatant was applied to a HisTrap HP column (GE Healthcare) previously equilibrated with lysis buffer. The proteins were eluted with a continuous gradient over 40 column volumes of elution buffer (20 mM HEPES, pH 7.5, 500 mM NaCl, 10% (v/v) glycerol, 500 mM Imidazole). Subsequently, pure fractions were buffer exchanged into final buffer (20 mM HEPES, pH 7.5, 500 mM NaCl, 10% (v/v) glycerol, 2 mM TCEP) using a HiPrep Desalting column (GE Healthcare) and stored over night at 4°C in final buffer. The proteins were additionally purified by Size Exclusion Chromatography (Superose™ 6 Increase 10/30 GL, GE Healthcare) in final buffer, flash frozen in liquid nitrogen and stored at - 20°C. The purification of DDX21$_{Fl}$ consists of an extra step where the protein is buffer exchanged in buffer C (50 mM Na Phosphate pH 7.4, 200 mM NaCl), cut by adding 8 units of thrombin (GE Healthcare) per mg of protein overnight at 4°C, and further purified by anion exchange chromatography (Resource S, GE Healthcare), using a linear gradient to 1 M NaCl, before the final gel filtration step.

RNA and R-loop preparation

All the RNA and DNA oligos were purchased HPLC purified, desalted and lyophilized from Microsynth (Switzerland). All RNA substrates were labelled with fluorescein at their 5' end (5'-FAM). The sequences are as follows: ssRNA – 15mer-5'-FAM-GUUUCGGAUCGACGC-3'; dsRNA (15/30mer – 3' overhang): 5'-FAM-GUUUCGGAUCGACGC-3' + 5'-GCGUCGAUCCGAAACUAUACUUAAUUUUAA-3' and the RNA G-quadruplex Q2, 30mer from the 3′ UTR of the PITX1 messenger RNA (McRae et al., 2017)): 5'-FAM-GUUGGGGCGGGCGUUGGGUUUGGGGGGACG-3'. Each RNA was diluted in ultra-pure DNase/RNase-free distilled water (Invitrogen) to a final concentration of 100 µM. For preparing stocks for the experiments, the RNAs were further dissolved to 10 µM in FP buffer (50 mM Tris pH 7.5, 50 mM KCl, 5 mM $MgCl_2$), and annealed by heating at 95°C for 5 minutes and slowly cooling to room temperature. These stocks were aliquoted and stored at -20°C. For the dsRNA substrate, the 15mer 5'-FAM labelled oligonucleotide was mixed with equimolar amounts of the 30mer RNA oligonucleotide, resulting in a 3' overhang dsRNA. To form the R-loop substrate, the labelled 15-mer RNA oligo (5'-FAM-GUUUCGGAUCGACGC-3') was annealed with DNA oligo 1 (5′-GTA CCCG GGGATCCTCTAGAGTCGAGCGTCGATCCG AAACTT GGCACTGGCCGTCGTTTTACAAC-3′) and DNA oligo 2 (5′-GTTGTAAAACGACGGCCAGTGCCTTTTCCCAGCCTCAATCTCATCACTCTAG AGGATCCCCGGGTAC-3′) following the procedure described in (Song et al., 2017).

SEC-MALS

The molecular weights of the constructs were determined by size exclusion chromatography coupled to multi angle light scattering (SEC-MALS). The mass measurements were performed on a Dionex UltiMate3000 HPLC system equipped with a 3 angles miniDAWN TREOS static light scattering detector (Wyatt Technology). The sample volumes of 100 µl at a concentration of 5 to 10 mg/mL, were applied to a Superose 6 10/300 GL column (GE Healthcare) previously equilibrated with 20 mM HEPES pH

7.5, 300 mM NaCl, 1 mM TCEP at a flow rate of 0.5 mL/min. The data were analysed using the ASTRA 6.1 software package (Wyatt technology), using the absorbance at 280 nm and the theoretical extinction coefficient for concentration measurements.

Circular Dichroism

Spectra of the DDX21 variants (at 5 µM in 50 mM Tris pH 7.5, 50 mM KCl, 5 mM $MgCl_2$) were collected with a J-815 CD Spectrometer (JASCO, Japan), (1 mm path length cuvette, 20°C, 260-200 nm). Thermal denaturation curves were acquired by heating the sample from 20 to 98°C, collecting CD data at 222 nm every two degrees. Spectra for the RNA G-quadruplex Q2 was measured at 20 µM in the same conditions except that the wavelength range was 320-220 nm.

Thermal difference spectra

UV/VIS absorbance spectra of the RNA molecules (20 µM in 50 mM Tris pH 7.5, 50 mM KCl, 5 mM $MgCl_2$) were collected with a J-815 CD Spectrometer (JASCO, Japan), (1 mm path length cuvette, 320-220 nm) and background corrected against spectra of buffer alone. Thermal difference spectra (TDS) were generated by subtracting buffer-corrected spectra at 20°C from those at 98°C. For direct comparison between Q2 RNA and ssRNA, the spectra were normalised.

Dynamic Light Scattering

Dynamic Light Scattering (DLS) data were acquired using a Stunner instrument (Unchained Labs) using a standard plate, with 10x10sec acquisitions at 25°C. The RNA Q2 concentration was 10 µM in 50 mM Tris pH 7.5, 50 mM KCl, 5 mM $MgCl_2$.

Mass photometry

All mass photometry measurements were executed on a Refeyn OneMP instrument. The calibration was done with a native marker protein standard mix (NativeMark Unstained Protein Standard, Thermo Scientific), which contains proteins ranging from 20 to 1200 kDa. Coverslips (24x50mm, No. 1.5H, Marienfeld) were cleaned by sequential sonication in Milli-Q water, isopropanol and Milli-Q-water, followed by drying with nitrogen. For each acquisition 2 µL of protein/RNA solution was applied to 18 µL buffer (50 mM Tris pH 7.5, 50 mM KCl, 5 mM $MgCl_2$) in a gasket (CultureWellTM Reusable Gasket, Grace Bio-Labs) on a coverslip. Typical working concentrations were 15 to 30 nM. Movies were recorded at 999 Hz with an exposure time of 0.95 ms by using the AcquireMP software. All mass photometry movies were processed and analysed in the DiscoverMP software. Protein samples and R-loop only were measured in duplicates and the R-loop-protein complex in triplicate (**Figures 2 and S6**).

Crosslinking

An aliquot of the corresponding DDX21 variant was thawed and buffer exchanged using PD-10 Desalting columns (GE Healthcare UK Limited) into 20 mM sodium phosphate, 500 mM NaCl, pH 7.6 at 4°C. It was diluted to 0.4 mg/mL and was mixed with 1mM BS^3 (freshly prepared in 20 mM sodium phosphate pH 7.6) and incubated 10 min at room temperature before stopping the reaction by the addition of 50 mM Tris pH 7.6. For glutaraldehyde crosslinking, the protocol was the same, with protein concentration of 0.1 mg/mL, glutaraldehyde concentration of 0.1% and incubation time of 5 min.

Analytical Ultra Centrifugation

Sedimentation equilibrium measurements were performed using a Beckman XL-A ultracentrifuge equipped with absorbance and interference optics. Data was acquired using a sample volume of 140

μL at a concentration of 20 μM in 20 mM Hepes pH 7.5, 300 mM NaCl at 20°C and a rotor speed (An60-Ti) of 5000 rpm after equilibrium was attained. Partial specific volumes and buffer densities were estimated using SEDNTERP (Laue, T., Shaw, B. D., Ridgeway, T. M., and Pelletier, S. L, 1992) and data were analysed using SEDPHAT (Vistica et al., 2004).

Fluorescence Polarization assays for RNA binding affinity measurements

Concentrated DDX21 protein stocks were buffer exchanged into FP buffer (50 mM Tris pH 7.5, 50 mM KCl, 5 mM $MgCl_2$) and diluted to the highest concentration used in the binding assay, 5 or 10 μM, depending on the construct and the RNA substrate. This high concentrated sample was diluted in FP buffer in 1/2 series to the lowest chosen concentration. The 1/2 series with a different starting concentration was used when more data points were required. The RNA stocks were diluted to 10 nM in FP buffer. 90 μL of RNA were mixed with 90 μL of the protein dilution series directly in the 96-well plate (Greiner 96 well flat bottom black polystyrene plates (Sigma-Aldrich)) and incubated for 5 min. Samples were excited at 485 nm, and fluorescence was detected at 535 nm. FP data were collected in an Infinite® F500 (Tecan) plate reader at 22° C. Three independent titration curves were measured for each complex, which were used to extract the average FP value and the standard errors at each titration point. The Tecan I-control software calculated the FP data. These were normalized and fit to the equation “Specific binding with Hill slope” (non-linear regression analysis) in GraphPad Prism: Y = Bmax*X^h/(K_d^h + X^h), where Y is the normalised FP signal, B_{max} is the maximum signal, X is the protein concentration, K_d is the protein concentration needed to achieve a half-maximum binding at equilibrium and corresponds to the equilibrium binding constant if h = 1.

Helicase assay and RNase T1 cleavage

The dsRNA substrate was diluted to 500 nM in 25 mM Hepes pH 7.5, 2 mM $MgCl_2$. The DDX21 variants were used directly from frozen stocks in final buffer. Reaction volumes were 30 μl, with 100 nM dsRNA, 2.5 μM DDX21, 2 mM ATP and 0.025 U/μl of RNase T1 (Thermo Scientific, 1000 U/μl frozen stock) in 25 mM Hepes pH 7.5, 2 mM $MgCl_2$, 33 mM NaCl. Unwinding/cleavage reactions were performed at 37°C and samples were taken after 5, 10, 15 and 30 min of incubation after the addition of ATP. Reactions were stopped by adding equal volumes of 2x loading buffer (20% glycerol, 0.8% SDS, 10 mM EDTA, 0.04% bromophenol blue) and the samples were separated in native 12% polyacrylamide TBE gels at 4°C.

RNA G-quadruplex remodelling and RNase T1 cleavage

The RNA G-quadruplex Q2 substrate was diluted to 500 nM in 50 mM Tris pH 7,5, 50 mM KCl, 2 mM $MgCl_2$, 10% glycerol. The DDX21 variants were used directly from frozen stocks in final buffer. Reaction volumes were 30 μl, with 100 nM Q2 RNA, 2.5 μM DDX21, 2 mM ATP and 0.0125 U/μl of RNase T1 (Thermo Scientific, 1000 U/μl frozen stock) in 50 mM Tris pH 7,5, 50 mM KCl, 25 mM NaCl, 2 mM $MgCl_2$, 10% glycerol. Remodelling/cleavage reactions were performed at 30°C and samples were taken after 20 min of incubation after the addition of ATP. Reactions were stopped by adding equal volumes of 2x loading buffer (20% glycerol, 0.8% SDS, 10 mM EDTA, 0.04% bromophenol blue) and heating at 95°C for 5 min to improve the sharpness of the bands. The samples were separated in native 12% polyacrylamide TBE gels at 4°C.

Homology modelling

The human $DDX21_{Fl}$ sequence was submitted to the online platform SwissModel in order to search for structural templates. Hits were evaluated for good overall sequence similarity and sequence coverage. The main hits were the bacterial DEAD-box helicases Hera (PDB IDs: 4KBF, 3I32) and CshA (PDB ID: 5IVL). For creating templates for the HC and DD domains (residues 186-621), Hera was chosen with 43% sequence identity over this range. With the individual domain models in hand, four models were created (**Figure S17**)), combining the two open conformations of the helicase core observed in Hera (PDB ID: 4KBF, chains a and b) and two positions of the helicase core with respect to the dimerization domain: one creating an “inverted V-shaped” (non-flat) structure as seen in the CshA crystal structure (PDB ID: 5IVL) and another where these domains are coplanar with the DD (flat conformation) (Core2-DD-RBD from Hera PDB ID: 3I32 and Core1 position from 4KBF). The NMR structure of the GUCT

domain (PDB ID: 2M3D) was also used to create these four models that cover a total of 524 residues (186-710). For fitting the models to the SAXS scattering curves, the N- and/or C-terminal unstructured tags were created using Pymol or I-TASSER (Yang et al., 2015). During revision, the crystal structures of the DDX21 HC were published (Chen et al., 2020) and these were used to modify the existing models. We superimposed each RecA-like domain separately into the $DDX21_{Core-DD}$ and $DDX21_{\Delta NC}$ models (RMSD of D1 (residues 188-397) and D2 (residues 410-563) RecA-like domains with those in the $DDX21_{Core-DD}$ was ~4 and with those in the $DDX21_{\Delta NC}$ was ~3.5) to place them in the optimised position fitting the SAXS data and then merge them to the DD/GUCT part of the models. Residues 529-533 were slightly remodelled to avoid clashes. Models were assessed and constructed using Coot (Emsley and Cowtan, 2004), PyMol (Schrödinger, LLC) and Chimera (Pettersen et al., 2004). Sequence alignment figures were made using Clustal Omega (Madeira et al., 2019) and Espript (Robert and Gouet, 2014).

Small-Angle X-Ray Scattering data collection and analysis

The data were collected at the European Synchrotron Radiation Facility (ESRF), beamline 29 (BM29) at a wavelength of 0.99 Å with a sample to detector distance of 2.867 m and a PILATUS 1M detector, covering a momentum transfer of 0.0025 < s > 0.5 $Å^{-1}$ [$s = 4\pi \sin(\theta)/\lambda$]. Measurements were made at 20°C. The proteins were analysed by size-exclusion chromatography in line with small-angle X-Ray scattering (SEC-SAXS) to avoid the signal from possible aggregates (with the exception of the $DDX21_{\Delta N}$ monomer mutant that was collected in batch mode). The samples were applied to a Superose 6 10/300 GL column (GE Healthcare) at a concentration of 9-12 mg/mL and run at a flow rate of 0.5 mL/min in 20 mM HEPES, pH 7.5, 500 mM NaCl, 10% (v/v) glycerol, 2 mM TCEP. During the elution, 3000 scattering measurements were taken with 1s time-frames. The *in-house* software BsxCuBE (Biosaxs Customized Beamline Environment) connected to a data processing pipeline (EDNA) (Incardona et al., 2009) was used to control the real time data display (two dimensional and one dimensional) and to provide the first automatic data processing up to a preliminary *ab initio* model. SAXS data were analysed using the ATSAS package version 2.8.3 (Franke et al., 2017) and ScÅtter (www.bioisis.net). For each sample, using Chromixs (Panjkovich and Svergun, 2018), an elution profile was generated with the integrated intensities plotted versus the recorded frame number. Using Chromixs, ~30 buffer frames were averaged and used to (i) subtract the buffer average from each frame of the sample peak selected and (ii) calculate the corresponding Radius of Gyration (R_g). The subtracted peak region was selected in Chromixs and averaged to generate the final scattering curve used for subsequent analysis. The scattering curves were initially viewed in PRIMUS (Konarev et al., 2003) where the R_g was obtained from the slope of the Guinier plot within the region defined by $s_{min} < s < s_{max}$ where $s_{max} < 1.3/R_g$ and s_{min} is the lowest angle data point included by the program (**Figure S13**). The P(r) function, the distribution of the intra-atomic distances (r) in the particle, was generated using the indirect transform program GNOM (Svergun, 1992) using a scattering range of s_{min}/s_{max} of 0.0072/0.3792 (Fl), 0.0075/0.3787 (ΔN), 0.0117/0.3788 (ΔNC), 0.0098/0.3787 (Core-DD) and 0.0135/0.3801 (ΔN monomer). The maximum distance (D_{max}) was selected by letting the P(r) curve decay smoothly to zero (**Table S3**). The R_g was also estimated from the P(r) function, such that, unlike the Guinier R_g estimation, the P(r) R_g calculation takes a larger scattering range into account. DATPOROD, DATMOW and DATVC within the ATSAS package (Fischer et al., 2010; Franke et al., 2017; Rambo and Tainer, 2013) were used to estimate the Porod Volume (Vp) and the concentration-independent estimate of the MW for the proteins (**Table S3**). The final figures were generated using PyMOL (Schrödinger, LLC) and Chimera (Pettersen et al., 2004).

To better address the flexibility of DDX21, we performed two types of analysis: EOM 2.1 analysis (ensemble optimization method) (Bernadó et al., 2007; Tria et al., 2015) (**Figure S16**) and nonlinear Cartesian Normal Mode Analysis (NOLB NMA) (Hoffmann and Grudinin, 2017) (**Figures 5** and **S17**). In the EOM procedure, a pool of 10,000 random structures of the $DDX21_{\Delta NC}$ dimer was generated by creating flexible linkers between the domains provided. These were the DD, that was kept fixed as the dimer interface and the HC and the GUCT domains, as three independent domains. Then, 100 cycles of genetic algorithm were run using all the data range. The genetic algorithm optimized the size of the ensemble and the fraction of occupancy of each conformer in the ensemble by minimizing the discrepancy between the calculated scattering curve and the experiential data (χ^2).

In the NOLB NMA procedure, the flat and “inverted V-shaped” $DDX21_{\Delta NC}$ models described above were flexibly fitted to the SAXS data through a χ^2 –minimizing optimization procedure based on the nonlinear

Cartesian NMA method called NOLB (Hoffmann and Grudinin, 2017) and a novel SAXS profile calculator called Pepsi-SAXS (Grudinin et al., 2017). More precisely, for each initial model we performed 100 optimization iterations. Each iteration comprised the computation of 60 slowest normal modes (using the NOLB tool), nonlinear structure deformation along these modes, and choosing the deformation with the least χ^2 value to the experimental scattering profile (using Pepsi-SAXS). A steepest-descent minimization algorithm was used at the end of each iteration to keep the local topology (bonds and angles) in agreement with the initial structure. The flexible fitting method is available as a standalone executable called Pepsi-SAXS-NMA at (https://team.inria.fr/nano-d/software/pepsi-saxs/). The resulting structures converged to low values of χ^2 (**Figures 5 and S17**).

Supplemental References

Ariyo, E.O., Booy, E.P., Patel, T.R., Dzananovic, E., McRae, E.K., Meier, M., McEleney, K., Stetefeld, J., and McKenna, S.A. (2015). Biophysical Characterization of G-Quadruplex Recognition in the PITX1 mRNA by the Specificity Domain of the Helicase RHAU. PLoS ONE *10*, e0144510.

Bernadó, P., Mylonas, E., Petoukhov, M.V., Blackledge, M., and Svergun, D.I. (2007). Structural Characterization of Flexible Proteins Using Small-Angle X-ray Scattering. Journal of the American Chemical Society *129*, 5656–5664.

Chen, Z., Li, Z., Hu, X., Xie, F., Kuang, S., Zhan, B., Gao, W., Chen, X., Gao, S., Li, Y., et al. (2020). Structural Basis of Human Helicase DDX21 in RNA Binding, Unwinding, and Antiviral Signal Activation. Adv Sci (Weinh) *7*, 2000532.

Drozdetskiy, A., Cole, C., Procter, J., and Barton, G.J. (2015). JPred4: a protein secondary structure prediction server. Nucleic Acids Res. *43*, W389-394.

Emsley, P., and Cowtan, K. (2004). Coot: model-building tools for molecular graphics. Acta Crystallogr. D Biol. Crystallogr. *60*, 2126–2132.

Fischer, H., Oliveira Neto, M. de, Napolitano, H.B., Polikarpov, I., and Craievich, A.F. (2010). Determination of the molecular weight of proteins in solution from a single small-angle X-ray scattering measurement on a relative scale. J Appl Cryst *43*, 101–109.

Franke, D., Petoukhov, M.V., Konarev, P.V., Panjkovich, A., Tuukkanen, A., Mertens, H.D.T., Kikhney, A.G., Hajizadeh, N.R., Franklin, J.M., Jeffries, C.M., et al. (2017). ATSAS 2.8: a comprehensive data analysis suite for small-angle scattering from macromolecular solutions. J Appl Cryst *50*, 1212–1225.

Grudinin, S., Garkavenko, M., and Kazennov, A. (2017). Pepsi-SAXS: an adaptive method for rapid and accurate computation of small-angle X-ray scattering profiles. Acta Crystallogr D Struct Biol *73*, 449–464.

Hardin, J.W., Hu, Y.X., and McKay, D.B. (2010). Structure of the RNA binding domain of a DEAD-box helicase bound to its ribosomal RNA target reveals a novel mode of recognition by an RNA recognition motif. J. Mol. Biol. *402*, 412–427.

Hoffmann, A., and Grudinin, S. (2017). NOLB: Nonlinear Rigid Block Normal-Mode Analysis Method. J Chem Theory Comput *13*, 2123–2134.

Huen, J., Lin, C.-L., Golzarroshan, B., Yi, W.-L., Yang, W.-Z., and Yuan, H.S. (2017). Structural Insights into a Unique Dimeric DEAD-Box Helicase CshA that Promotes RNA Decay. Structure *25*, 469–481.

Incardona, M.-F., Bourenkov, G.P., Levik, K., Pieritz, R.A., Popov, A.N., and Svensson, O. (2009). EDNA: a framework for plugin-based applications applied to X-ray experiment online data analysis. J Synchrotron Rad, J Synchrotron Radiat *16*, 872–879.

Klostermeier, D. (2013). Rearranging RNA structures at 75°C? Toward the molecular mechanism and physiological function of the Thermus thermophilus DEAD-box helicase Hera. Biopolymers *99*, 1137–1146.

Konarev, P.V., Volkov, V.V., Sokolova, A.V., Koch, M.H.J., and Svergun, D.I. (2003). PRIMUS: a Windows PC-based system for small-angle scattering data analysis. J Appl Cryst, J Appl Crystallogr *36*, 1277–1282.

Laue, T., Shaw, B. D., Ridgeway, T. M., and Pelletier, S. L (1992). Analytical Ultracentrifugation in Biochemistry and Polymer Science. (The Royal Society of Chemistry, Cambridge, U.K.), pp. 90–125.

Linder, P., and Jankowsky, E. (2011). From unwinding to clamping - the DEAD box RNA helicase family. Nat. Rev. Mol. Cell Biol. *12*, 505–516.

Madeira, F., Park, Y.M., Lee, J., Buso, N., Gur, T., Madhusoodanan, N., Basutkar, P., Tivey, A.R.N., Potter, S.C., Finn, R.D., et al. (2019). The EMBL-EBI search and sequence analysis tools APIs in 2019. Nucleic Acids Res. *47*, W636–W641.

McRae, E.K.S., Booy, E.P., Moya-Torres, A., Ezzati, P., Stetefeld, J., and McKenna, S.A. (2017). Human DDX21 binds and unwinds RNA guanine quadruplexes. Nucleic Acids Res. *45*, 6656–6668.

Mergny, J.-L., Li, J., Lacroix, L., Amrane, S., and Chaires, J.B. (2005). Thermal difference spectra: a specific signature for nucleic acid structures. Nucleic Acids Res. *33*, e138.

Ohnishi, S., Pääkkönen, K., Koshiba, S., Tochio, N., Sato, M., Kobayashi, N., Harada, T., Watanabe, S., Muto, Y., Güntert, P., et al. (2009). Solution structure of the GUCT domain from human RNA helicase II/Gu beta reveals the RRM fold, but implausible RNA interactions. Proteins *74*, 133–144.

Panjkovich, A., and Svergun, D.I. (2018). CHROMIXS: automatic and interactive analysis of chromatography-coupled small-angle X-ray scattering data. Bioinformatics *34*, 1944–1946.

Pettersen, E.F., Goddard, T.D., Huang, C.C., Couch, G.S., Greenblatt, D.M., Meng, E.C., and Ferrin, T.E. (2004). UCSF Chimera--a visualization system for exploratory research and analysis. J Comput Chem *25*, 1605–1612.

Rambo, R.P., and Tainer, J.A. (2013). Accurate assessment of mass, models and resolution by small-angle scattering. Nature *496*, 477–481.

Robert, X., and Gouet, P. (2014). Deciphering key features in protein structures with the new ENDscript server. Nucleic Acids Res. *42*, W320-324.

Song, C., Hotz-Wagenblatt, A., Voit, R., and Grummt, I. (2017). SIRT7 and the DEAD-box helicase DDX21 cooperate to resolve genomic R loops and safeguard genome stability. Genes Dev. *31*, 1370–1381.

Steimer, L., Wurm, J.P., Linden, M.H., Rudolph, M.G., Wöhnert, J., and Klostermeier, D. (2013). Recognition of two distinct elements in the RNA substrate by the RNA-binding domain of the T. thermophilus DEAD box helicase Hera. Nucleic Acids Res. *41*, 6259–6272.

Svergun, D.I. (1992). Determination of the regularization parameter in indirect-transform methods using perceptual criteria. J Appl Cryst, J Appl Crystallogr *25*, 495–503.

Tria, G., Mertens, H.D.T., Kachala, M., and Svergun, D.I. (2015). Advanced ensemble modelling of flexible macromolecules using X-ray solution scattering. IUCrJ *2*, 207–217.

Valdez, B.C., Perlaky, L., and Henning, D. (2002a). Expression, cellular localization, and enzymatic activities of RNA helicase II/Gu(beta). Exp. Cell Res. *276*, 249–263.

Valdez, B.C., Yang, H., Hong, E., and Sequitin, A.M. (2002b). Genomic structure of newly identified paralogue of RNA helicase II/Gu: detection of pseudogenes and multiple alternatively spliced mRNAs. Gene *284*, 53–61.

Vistica, J., Dam, J., Balbo, A., Yikilmaz, E., Mariuzza, R.A., Rouault, T.A., and Schuck, P. (2004). Sedimentation equilibrium analysis of protein interactions with global implicit mass conservation constraints and systematic noise decomposition. Anal. Biochem. *326*, 234–256.

Xu, L., Wang, L., Peng, J., Li, F., Wu, L., Zhang, B., Lv, M., Zhang, J., Gong, Q., Zhang, R., et al. (2017). Insights into the Structure of Dimeric RNA Helicase CsdA and Indispensable Role of Its C-Terminal Regions. Structure *25*, 1795-1808.e5.

Yang, J., Yan, R., Roy, A., Xu, D., Poisson, J., and Zhang, Y. (2015). The I-TASSER Suite: protein structure and function prediction. Nat. Methods *12*, 7–8.
